# Copper-catalyzed CuAAC/intramolecular C–H arylation sequence: Synthesis of annulated 1,2,3-triazoles

**DOI:** 10.3762/bjoc.8.202

**Published:** 2012-10-16

**Authors:** Rajkumar Jeyachandran, Harish Kumar Potukuchi, Lutz Ackermann

**Affiliations:** 1 Institut für Organische und Biomolekulare Chemie, Georg-August-Universität, Tammannstrasse 2, 37077 Göttingen, Germany; 2 Lehrstuhl für Organische Chemie I, Lichtenbergstrasse 4, 85747 Garching, Germany

**Keywords:** cascade reaction, C–H functionalization, copper, heteroarenes, triazoles

## Abstract

Step-economical syntheses of annulated 1,2,3-triazoles were accomplished through copper-catalyzed intramolecular direct arylations in sustainable one-pot reactions. Thus, catalyzed cascade reactions involving [3 + 2]-azide–alkyne cycloadditions (CuAAC) and C–H bond functionalizations provided direct access to fully substituted 1,2,3-triazoles with excellent chemo- and regioselectivities. Likewise, the optimized catalytic system proved applicable to the direct preparation of 1,2-diarylated azoles through a one-pot C–H/N–H arylation reaction.

## Introduction

**Scheme 1:**
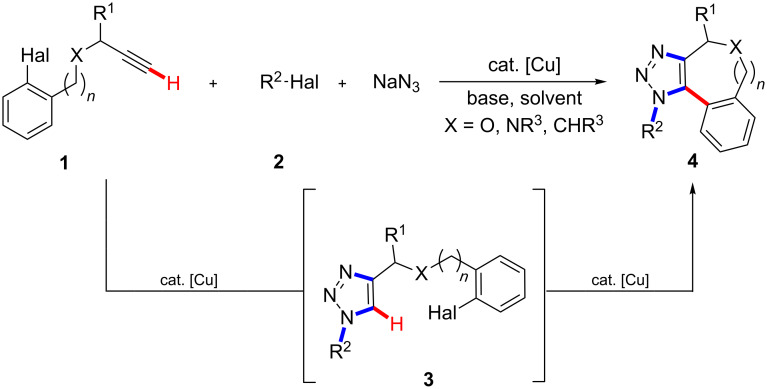
Copper-catalyzed step-economical C–H arylation-based cascade reaction.

Transition-metal-catalyzed C–H bond functionalizations are increasingly viable tools for step-economical syntheses of various valuable bioactive compounds [1–3], which avoid the preparation and use of preactivated substrates [4–16]. This streamlining of organic synthesis has predominantly been accomplished with palladium [4–16], rhodium [17–19] or ruthenium [20–22] complexes [4–16]. However, less expensive nickel, cobalt, iron or copper catalysts bear great potential for the development of economically attractive transformations [23–50]. In this context, we previously reported on the use of cost-effective copper(I) catalysts for direct arylations of 1,2,3-triazoles. Thus, we showed that intermolecular copper-catalyzed C–H bond functionalizations could be combined with the Huisgen [51] copper(I)-catalyzed [52–53] [3 + 2]-azide–alkyne cycloaddition (CuAAC)[54], while C–H bond arylations of 1,2,3-triazoles were previously only accomplished with more expensive palladium [55–62] or ruthenium [63–66] catalysts. Notably, this strategy allowed for the atom-economical synthesis of fully substituted 1,2,3-triazoles in a highly regioselective fashion [54,67]. While the research groups of Rutjes [68] as well as Sharpless [69] elegantly devised alternative approaches exploiting 1-haloalkynes [70], we became interested in exploring a single [71–73] inexpensive copper catalyst for one-pot reaction sequences comprising a 1,3-dipolar cycloaddition along with an intramolecular C–H bond arylation; in particular, because of the notable biological activities exerted by fully substituted 1,2,3-triazoles [74–88]. As a consequence, we wish to present herein novel cascade reactions, in which cost-effective copper(I) compounds serve as the catalyst for two mechanistically distinct transformations for the synthesis of fully substituted annulated 1,2,3-triazoles as well as for twofold N–H/C–H bond arylations. Notable features of our strategy include (i) the development of a chemoselective C–H arylation-based three-component reaction, as well as (ii) the use of inexpensive CuI for the formation of up to one C–C and three C–N bonds in a site-selective fashion (Scheme 1).

## Results and Discussion

We initiated our studies by exploring reaction conditions for the key copper-catalyzed intramolecular direct C–H bond arylation, employing substrate **3a** (Table 1). Notably, the envisioned C–H bond functionalization occurred readily with the aryl iodide **3a** when catalytic amounts of CuI were used, even at a reaction temperature as low as 60 °C, with optimal yields being obtained at 80 °C (Table 1, entries 1–6). While the transformation proceeded efficiently with LiO*t*-Bu as the stoichiometric base, K_3_PO_4_ only led to unsatisfactory results, even when additional stabilizing ligands were used (Table 1, entries 7–10).

**Table 1:** Optimization studies for the intramolecular direct arylation of triazole **3a**.^a^

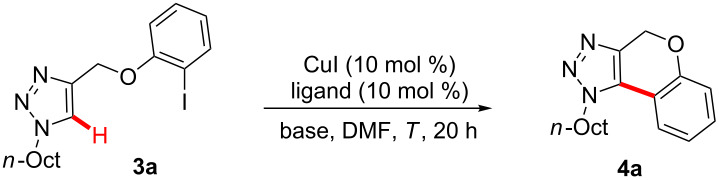				

entry	base	ligand	*T* [°C]	isolated yield [%]

1	LiO*t*-Bu	–	140	82
2	LiO*t*-Bu	–	120	97
3	LiO*t*-Bu	–	100	91

**4**	**LiO*t*-Bu**	–	**80**	**93**

5	LiO*t*-Bu	–	60	72
6	LiO*t*-Bu	–	20	<2^b^
7	K_3_PO_4_	DMEDA	140	5^b^
8	K_3_PO_4_	*N*,*N*-dimethylglycine	140	5^b^
9	K_3_PO_4_	2,2-bipyridyl	140	4^b^
10	K_3_PO_4_	1,10-phenanthroline	140	11

^a^General reaction conditions: **3a** (1.00 mmol), CuI (10 mol %), ligand (10 mol %), DMF (3.0 mL). ^b^By ^1^H NMR spectroscopy.

**Scheme 2:**
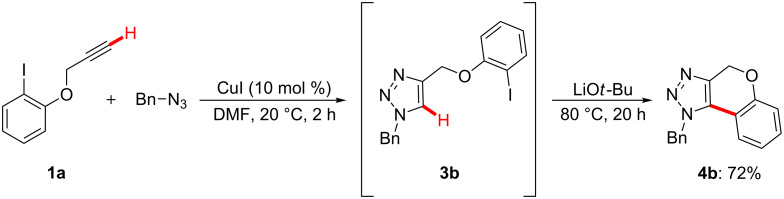
Copper-catalyzed sequential catalysis with alkyne **1a**.

With optimized reaction conditions for the intramolecular direct arylation in hand, we tested the possibility of its implementation in a sequential synthesis of 1,4-dihydrochromeno[3,4-*d*][1,2,3]triazole (**4b**, Scheme 2). We were delighted to observe that the desired reaction sequence consisting of a copper-catalyzed 1,3-dipolar cycloaddition and an intramolecular C–H bond arylation converted alkyne **1a** to the desired product **4b** with high catalytic efficacy.

**Scheme 3:**
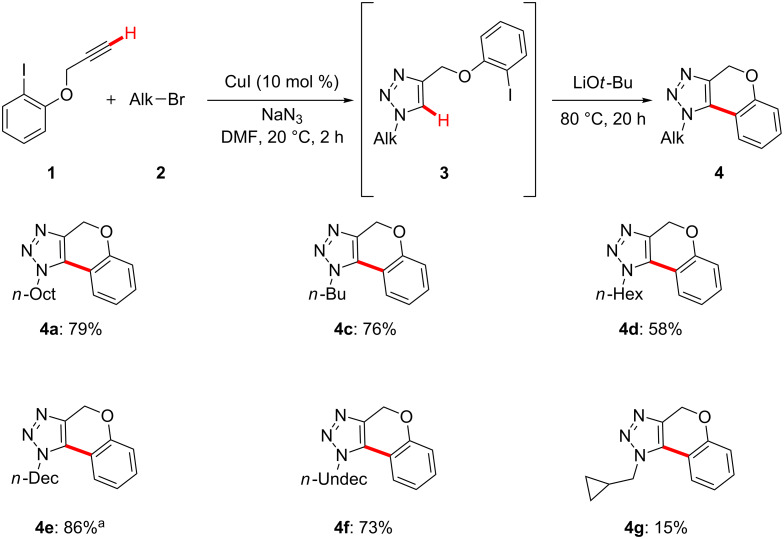
Copper-catalyzed reaction sequence using alkyl bromides **2**. General reaction conditions: **1** (1.00 mmol), **2** (1.00 mmol), NaN_3_ (1.05 mmol), CuI (10 mol %), DMF (3.0 mL), LiO*t*-Bu (2.00 mmol); yields of isolated product. ^a^60 °C in the first step.

Subsequently, we explored the extension of this approach to the development of a chemoselective three-component one-pot reaction. Thus, we found that alkyl bromides **2** could be directly employed as user-friendly substrates for the in situ formation of the corresponding organic azides (Scheme 3). Notably, the catalytic system proved broadly applicable, and a variety of organic electrophiles **2**, thereby, delivered differently decorated *N*-substituted 1,4-dihydrochromeno[3,4-*d*][1,2,3]triazoles **4**.

**Scheme 4:**
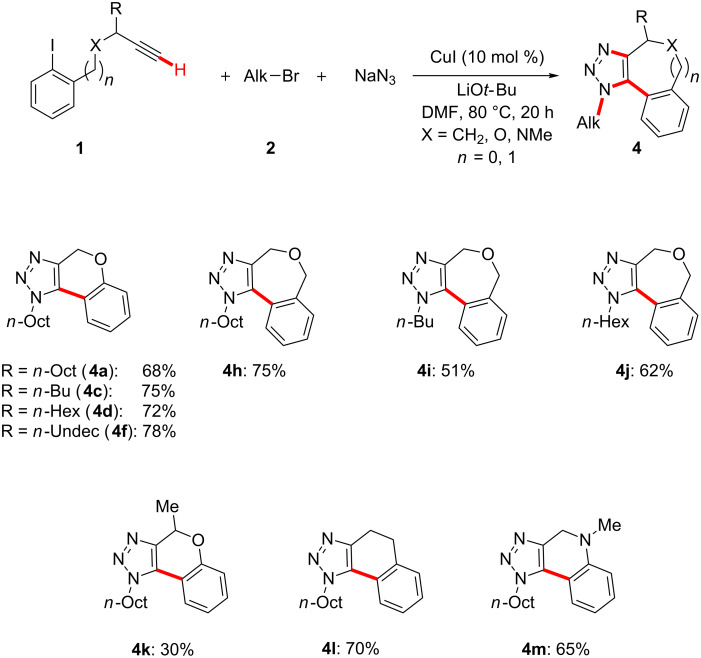
Nonsequential cascade synthesis of fully substituted triazoles **4**. General reaction conditions: **1** (1.00 mmol), **2** (1.00 mmol), NaN_3_ (1.05 mmol), CuI (10 mol %) DMF (3.0 mL), LiO*t*-Bu (2.00 mmol); yields of isolated product.

Importantly, performing the one-pot reaction in a sequential fashion was not found to be mandatory. Indeed, our strategy turned out to be viable in a nonsequential manner by directly employing equimolar amounts of the three substrates. Hence, inexpensive CuI allowed the direct assembly of aryl iodides **1**, alkyl bromides **2** and NaN_3_ with excellent chemo- and regioselectivities (Scheme 4). Thereby, a variety of annulated 1,2,3-triazoles **4** were obtained, featuring six- or seven-membered rings as key structural motifs. It is particularly noteworthy that the copper-catalyzed transformation enabled the formation of one C–C and three C–N bonds in a chemoselective manner, and thereby provided atom- and step-economical access to annulated carbo- as well as O- and N-heterocycles.

**Scheme 5:**
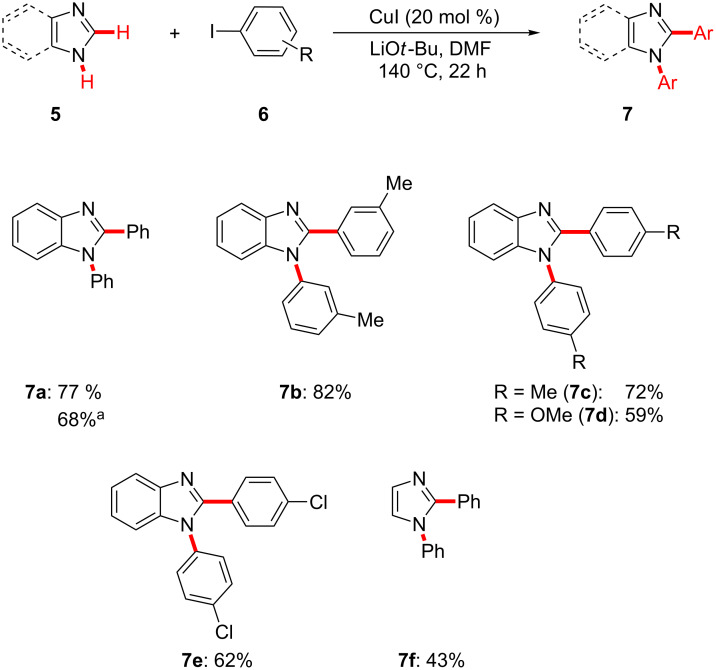
Copper-catalyzed one-pot twofold C–H/N–H arylation with azoles **5**. ^a^Reaction performed at 120 °C.

Finally, we found that the catalytic system also proved to be applicable to the one-pot copper-catalyzed direct arylation of various azoles **5** through N–H/C–H bond cleavages with aryl iodides **6** as the organic electrophiles (Scheme 5).

## Conclusion

In summary, we have reported on the use of inexpensive copper(I) complexes for step- and atom-economical sequential catalytic transformations involving direct C–H bond arylations. Thus, CuI enabled the synthesis of fully substituted 1,2,3-triazoles through cascade reactions consisting of copper(I)-catalyzed [3 + 2]-azide–alkyne cycloadditions (CuAAC) and intramolecular C–H bond arylations. Notably, the optimized copper catalyst accelerated two mechanistically distinct transformations, which set the stage for the formation of up to one C–C and three C–N bonds in a chemo- and regioselective fashion, and also allowed for twofold C–H/N–H bond arylations on various azoles.

## Experimental

### General information

Catalytic reactions were carried out under an inert atmosphere of nitrogen using predried glassware. All chemicals were used as received without further purification unless otherwise specified. DMF was dried over CaH_2_. Alkynes **1** [89–92] and triazoles **3** [93] were synthesized according to previously described methods. CuI (99.999%) was purchased from ABCR with the following specifications: Ag <3 ppm, Ca = 2 ppm, Fe = 1 ppm, Mg <1 ppm, Zn <1 ppm. Yields refer to isolated compounds, estimated to be >95 % pure, as determined by ^1^H NMR. Thin-layer chromatography (TLC) was carried out on silica gel 60 F254 aluminum plates (Merck). Chromatography: Merck silica gel 60 (40–63 μm). NMR: Spectra were recorded on Varian Unity 300, Mercury 300 or Inova 500 in the solvent indicated; chemical shifts (δ) are given in parts per million (ppm). All IR spectra were taken on a Bruker FTIR Alpha device. MS: EIMS-spectra were recorded with Finnigan MAT 95, 70 eV; high-resolution mass spectrometry (HRMS) with APEX IV 7T FTICR, Bruker Daltonic. Melting points were determined with a Stuart melting-point apparatus SMP3, Barlworld Scientific; values are uncorrected.

#### General procedure for the synthesis of triazoles 4

NaN_3_ (1.05 equiv), CuI (10 mol %), LiO*t*-Bu (2.00 equiv), alkyne **1** (1.00 equiv) and alkyl bromide **2** (1.00 equiv) were dissolved in DMF (3.0 mL) and stirred at 80 °C for 20 h. Then, H_2_O (50 mL) was added at ambient temperature, and the resulting mixture was extracted with EtOAc (3 × 50 mL). The combined organic layers were washed with saturated aq NH_4_Cl (50 mL), H_2_O (50 mL) and brine (50 mL), dried over Na_2_SO_4_, filtered and concentrated in vacuo. The remaining residue was purified by column chromatography on silica gel (*n*-hexane/EtOAc).

## Supporting Information

Supporting Information containing all experimental details and analytical data of new compounds as well as their ^1^H and ^13^C spectra are provided.

File 1 Experimental procedures, characterization data, and NMR spectra for new compounds.

## References

[1] Seregin I V, Gevorgyan V (2007). Chem Soc Rev.

[2] Nakao Y (2011). Synthesis.

[3] Zhao D, You J, Hu C (2011). Chem–Eur J.

[4] (2012). Special Issue 6 "C-H Functionalization". Acc Chem Res.

[5] Hickman A J, Sanford M S (2012). Nature.

[6] Yeung C S, Dong V M (2011). Chem Rev.

[7] Ackermann L (2011). Chem Rev.

[8] McMurray L, O'Hara F, Gaunt M J (2011). Chem Soc Rev.

[9] Wencel-Delord J, Dröge T, Liu F, Glorius F (2011). Chem Soc Rev.

[10] Ackermann L (2010). Chem Commun.

[11] Sun C-L, Li B-J, Shi Z-J (2010). Chem Commun.

[12] Colby D A, Bergman R G, Ellman J A (2010). Chem Rev.

[13] Fagnou K (2010). Top Curr Chem.

[14] Boutadla Y, Davies D L, Macgregor S A, Poblador-Bahamonde A I (2009). Dalton Trans.

[15] Ackermann L, Vicente R, Kapdi A (2009). Angew Chem, Int Ed.

[16] Thansandote P, Lautens M (2009). Chem–Eur J.

[17] Zhu C, Wang R, Falck J R (2012). Chem–Asian J.

[18] Song G, Wang F, Li X (2012). Chem Soc Rev.

[19] Satoh T, Miura M (2010). Chem–Eur J.

[20] Ackermann L (2010). Pure Appl Chem.

[21] Ackermann L (2010). Isr J Chem.

[22] Ackermann L, Vicente R (2010). Top Curr Chem.

[23] Nakamura E, Yoshikai N (2010). J Org Chem.

[24] Su Y, Jia W, Jiao N (2011). Synthesis.

[25] Daugulis O (2010). Top Curr Chem.

[26] Yoshikai N (2011). Synlett.

[27] Kulkarni A, Daugulis O (2009). Synthesis.

[28] Cao H, Zhan H, Lin Y, Lin X, Du Z, Jiang H (2012). Org Lett.

[29] Das B, Reddy G C, Balasubramanyam P, Salvanna N (2012). Tetrahedron.

[30] Ciana C-L, Phipps R J, Brandt J R, Meyer F-M, Gaunt M J (2011). Angew Chem, Int Ed.

[31] Duong H A, Gilligan R E, Cooke M L, Phipps R J, Gaunt M J (2011). Angew Chem, Int Ed.

[32] Huang G, Sun H, Qiu X, Jin C, Lin C, Shen Y, Jiang J, Wang L (2011). Org Lett.

[33] Popov I, Lindeman S, Daugulis O (2011). J Am Chem Soc.

[34] Kawano T, Matsuyama N, Hirano K, Satoh T, Miura M (2010). J Org Chem.

[35] Barbero N, San Martin R, Dominguez E (2010). Org Biomol Chem.

[36] Pacheco Berciano B, Lebrequier S, Besselievre F, Piguel S (2010). Org Lett.

[37] Besselievre F, Piguel S (2009). Angew Chem, Int Ed.

[38] Fukuzawa S-I, Shimizu E, Ogata K (2009). Heterocycles.

[39] Kawano T, Yoshizumi T, Hirano K, Satoh T, Miura M (2009). Org Lett.

[40] Zhao D, Wang W, Yang F, Lan J, Yang L, Gao G, You J (2009). Angew Chem, Int Ed.

[41] Yotphan S, Bergman R G, Ellman J A (2009). Org Lett.

[42] Do H-Q, Daugulis O (2008). J Am Chem Soc.

[43] Yoshizumi T, Tsurugi H, Satoh T, Miura M (2008). Tetrahedron Lett.

[44] Do H-Q, Daugulis O (2007). J Am Chem Soc.

[45] Nishino M, Hirano K, Satoh T, Miura M (2012). Angew Chem, Int Ed.

[46] Do H-Q, Daugulis O (2011). J Am Chem Soc.

[47] Kitahara M, Umeda N, Hirano K, Satoh T, Miura M (2011). J Am Chem Soc.

[48] Zhu M, Fujita K-i, Yamaguchi R (2011). Chem Commun.

[49] Song W, Ackermann L (2012). Angew Chem, Int Ed.

[50] Ackermann L, Punji B, Song W (2011). Adv Synth Catal.

[51] Huisgen R (1963). Angew Chem.

[52] Tornoe C W, Christensen C, Meldal M (2002). J Org Chem.

[53] Rostovtsev V V, Green L G, Fokin V V, Sharpless K B (2002). Angew Chem, Int Ed.

[54] Ackermann L, Potukuchi H K, Landsberg D, Vicente R (2008). Org Lett.

[55] Chuprakov S, Chernyak N, Dudnik A S, Gevorgyan V (2007). Org Lett.

[56] Iwasaki M, Yorimitsu H, Oshima K (2007). Chem–Asian J.

[57] Ackermann L, Vicente R, Born R (2008). Adv Synth Catal.

[58] Ackermann L, Althammer A, Fenner S (2009). Angew Chem, Int Ed.

[59] Ackermann L, Vicente R (2009). Org Lett.

[60] Lapointe D, Fagnou K (2009). Org Lett.

[61] Schulman J M, Friedman A A, Panteleev J, Lautens M (2012). Chem Commun.

[62] Ackermann L, Jeyachandran R, Potukuchi H K, Novak P, Büttner L (2010). Org Lett.

[63] Ackermann L, Vicente R, Althammer A (2008). Org Lett.

[64] Ackermann L, Born R, Vicente R (2009). ChemSusChem.

[65] Ackermann L, Vicente R, Potukuchi H K, Pirovano V (2010). Org Lett.

[66] Ackermann L, Novák P, Vicente R, Pirovano V, Potukuchi H K (2010). Synthesis.

[67] Ackermann L, Potukuchi H K (2010). Org Biomol Chem.

[68] Kuijpers B H M, Dijkmans G C T, Groothuys S, Quaedflieg P J L M, Blaauw R H, van Delft F L, Rutjes F P J T (2005). Synlett.

[69] Hein J E, Tripp J C, Krasnova L B, Sharpless K B, Fokin V V (2009). Angew Chem, Int Ed.

[70] Spiteri C, Moses J E (2010). Angew Chem, Int Ed.

[71] Ackermann L, Born R, Álvarez-Bercedo P (2007). Angew Chem, Int Ed.

[72] Ackermann L, Althammer A (2007). Angew Chem, Int Ed.

[73] Ackermann L, Althammer A, Mayer P (2009). Synthesis.

[74] Astruc D, Liang L, Rapakousiou A, Ruiz J (2012). Acc Chem Res.

[75] Agalave S G, Maujan S R, Pore V S (2011). Chem–Asian J.

[76] Pedersen D S, Abell A (2011). Eur J Org Chem.

[77] Hänni K D, Leigh D A (2010). Chem Soc Rev.

[78] Kappe C O, Van der Eycken E (2010). Chem Soc Rev.

[79] El-Sagheer A H, Brown T (2010). Chem Soc Rev.

[80] Qin A, Lam J W Y, Tang B Z (2010). Chem Soc Rev.

[81] Meldal M, Tornoe C W (2008). Chem Rev.

[82] Nandivada H, Jiang X, Lahann J (2007). Adv Mater.

[83] Angell Y L, Burgess K (2007). Chem Soc Rev.

[84] Fournier D, Hoogenboom R, Schubert U S (2007). Chem Soc Rev.

[85] Moses J E, Moorhouse A D (2007). Chem Soc Rev.

[86] Lutz J-F (2007). Angew Chem, Int Ed.

[87] Dondoni A (2007). Chem–Asian J.

[88] Kolb H C, Sharpless K B (2003). Drug Discovery Today.

[89] Bowman W R, Krintel S L, Schilling M B (2004). Org Biomol Chem.

[90] Pastine S J, Youn S W, Sames D (2003). Org Lett.

[91] Wang R T, Chou F L, Luo F T (1990). J Org Chem.

[92] Shore G, Organ M G (2008). Chem–Eur J.

[93] Kacprzak K (2005). Synlett.

