# Supplementary material for: Copper-catalyzed CuAAC/intramolecular C–H arylation sequence: Synthesis of annulated 1,2,3-triazoles
Source: Beilstein J Org Chem. 2012 Oct 16;8:1771–7. doi: 10.3762/bjoc.8.202 (PMC3511011; doi:10.3762/bjoc.8.202)
Supplement: File 1 — Experimental procedures, characterization data, and NMR spectra for new compounds. [file Beilstein_J_Org_Chem-08-1771-s001.pdf]

Supporting Information

for

Copper-catalyzed CuAAC/intramolecular C–H arylation sequence: Synthesis of annulated 1,2,3-triazoles

Rajkumar Jeyachandran,[1] Harish Kumar Potukuchi,[1,2] and Lutz Ackermann[1]*

Address: [1]Institut für Organische und Biomolekulare Chemie, Georg-August-Universitaet, Tammannstrasse 2, 37077 Goettingen, Germany and [2]Lehrstuhl fuer Organische Chemie I, Lichtenbergstrasse 4, 85747 Garching, Germany.

Email: Lutz Ackermann* - lutz.ackermann@chemie.uni-goettingen.de

*Corresponding author

Experimental procedures, characterization data, and NMR spectra for new compounds.

General information…...…………………………………………………...…..….......S2
General procedure for the sequential synthesis of triazoles **4**..................................S2
Characterization data for compounds **4a**–**4g**..S3
General procedure for the one-pot synthesis of triazoles **4**......................................S8
Characterization data for compounds **4a**, **4c**–**4d**, **4f** and **4h**–**4m**.............................S8
General procedure for the copper-catalyzed one-pot C–C/C–N arylation
with azoles **5**...S14
Characterization data for compounds **7a**–**7f**..S14
References..S18
NMR-spectra..S20

General information

Catalytic reactions were carried out under an inert atmosphere of nitrogen using predried glassware. All chemicals were used as received without further purification unless otherwise specified. DMF was dried over CaH_2. Triazoles [1] **3** and alkynes [2] **1** were synthesized according to previously described methods. CuI (99.999 %) was purchased from ABCR with the following specifications: Ag <3 ppm, Ca 2 ppm, Fe 1 ppm, Mg <1 ppm, Zn <1 ppm. Yields refer to isolated compounds, estimated to be >95% pure as determined by ^{1}H NMR. Thin-layer chromatography (TLC) was carried out on silica gel 60 F254 aluminium plates (Merck). Detection under UV light at 254 nm. Chromatography: Merck silica gel 60 (40–63 µm). ^{1}H and ^{13}C NMR-spectra were recorded at 300 (^{1}H) and 75 {^{13}C, APT (Attached Proton Test)}, respectively, on Varian Unity-300 and AMX 300 instruments in $CDCl_3$ solutions, chemical shifts (δ) are given in ppm. All IR spectra were taken on a Bruker FTIR Alpha device. MS: EIMS-spectra were recorded with Finnigan MAT 95, 70 eV; High-resolution mass spectrometry (HRMS) with APEX IV 7T FTICR, Bruker Daltonic. Melting points were determined with a Stuart melting point apparatus SMP3, Barlworld Scientific; values are uncorrected.

General procedure for the sequential synthesis of triazoles 4

NaN_3 (1.05 equiv), CuI (10 mol %), 1-iodo-2-(prop-2-yn-1-yloxy)benzene (**1a**) (1.00 equiv) and alkyl bromide **2** (1.00 equiv) were dissolved in DMF (3.0 mL) and stirred at ambient temperature for 2 h. Thereafter, LiO*t*-Bu (2.00 equiv) was added under N_2 and the reaction mixture was stirred at 80 °C for 20 h. Then, H_2O (50 mL) was added at ambient temperature, and the resulting mixture was extracted with EtOAc (3 × 50 mL). The combined organic layers were washed with saturated aq NH_4Cl (50 mL), H_2O (50 mL) and brine (50 mL), dried over Na_2SO_4, filtered and

concentrated in vacuo. The remaining residue was purified by column chromatography on silica gel (*n*-hexane/EtOAc).

1-*n*-Octyl-1,4-dihydrochromeno[4,3-*d*][1,2,3]triazole (4a):

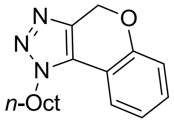

The general procedure was followed using NaN_3 (0.068 g, 1.05 mmol), CuI (19 mg, 0.100 mmol, 10 mol %), 1-iodo-2-(prop-2-yn-1-yloxy)benzene (**1a**, 0.256 g, 0.992 mmol), *n*-octyl bromide (0.203 g, 1.05 mmol) and LiO*t*-Bu (0.160 g, 2.00 mmol). Purification on silica gel (*n*-hexane/EtOAc = 10/1 → 5/1) gave **4a** (0.225 g, 79%) as a yellow oil. ^{1}H NMR (300 MHz, $CDCl_3$) δ 7.41 (dd, *J* = 8.0, 1.6 Hz, 1H), 7.30–7.23 (m, 1H), 7.08–7.01 (m, 2H), 5.44 (s, 2H), 4.58 (t, *J* = 7.4 Hz, 2H), 2.02–1.88 (m, 2H), 1.47–1.13 (m, 10H), 0.85 (t, *J* = 6.9 Hz, 3H); ^{13}C NMR (75 MHz, $CDCl_3$) δ 153.6 (C_q), 139.4 (C_q), 130.4 (CH), 126.9 (C_q), 122.1 (CH), 122.0 (CH), 118.1 (CH), 114.2 (C_q), 64.5 (CH_2), 49.9 (CH_2), 31.7 (CH_2), 29.7 (CH_2), 29.0 (CH_2), 28.9 (CH_2), 26.5 (CH_2), 22.6 (CH_2), 14.1 (CH_3); IR (film): 2954, 2926, 2855, 1519, 1464, 1446, 1335, 1200, 1123, 1022, 839 cm^{-1}; MS (EI) *m*/*z* (relative intensity) 285 (100) [M^+], 269 (6), 228 (10), 214 (30), 158 (50), 145 (34), 103 (12), 77 (8), 55 (10), 43 (18), 41 (26); HRMS (ESI) *m*/*z* calcd for $C_{17}H_{23}N_3O$ [$M+H^+$] 286.1914, found 286.1915. The spectral data were in accordance with those reported in the literature [3].

1-Benzyl-1,4-dihydrochromeno[3,4-*d*][1,2,3]triazole (4b):

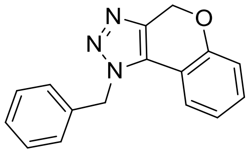

The general procedure was followed using benzyl azide (0.133 g, 1.00 mmol), CuI (19 mg, 0.100 mmol, 10 mol %), 1-iodo-2-(prop-2-yn-1-yloxy)benzene (**1a**, 0.259 g, 1.00 mmol) and LiO*t*-Bu (0.161 g, 2.01 mmol). Purification on silica gel (*n*-hexane/EtOAc = 10/1 → 5/1) gave **4b** (0.190 g, 72%) as a colorless solid. Mp = 143-144 °C; ^{1}H NMR (300 MHz, $CDCl_3$) δ 7.41–7.26 (m, 3H), 7.25–7.14 (m, 4H), 7.04–6.97 (m, 1H), 6.96–6.84 (m, 1H), 5.82 (s, 2H), 5.48 (s, 2H); ^{13}C NMR (75 MHz, $CDCl_3$) δ 153.6 (C_q), 139.7 (C_q), 134.6 (C_q), 130.5 (CH), 129.1 (CH), 128.3 (CH), 127.7 (C_q), 126.4 (CH), 122.5 (CH), 122.0 (CH), 117.9 (CH), 113.8 (C_q), 64.4 (CH_2), 53.0 (CH_2); IR (KBr): 2725, 2361, 1699, 1653, 1617, 1558, 1337, 1197, 1110, 1024, 848, 768 cm^{-1}; MS (EI) *m*/*z* (relative intensity): 263 (100) [M^+], 234 (34), 91 (14); HRMS (ESI) *m*/*z* calcd for $C_{16}H_{13}N_3O$ [$M+Na^+$] 286.0951, found 286.0945. The spectral data were in accordance with those reported in the literature [3].

1-*n*-Butyl-1,4-dihydrochromeno[4,3-*d*][1,2,3]triazole (4c):

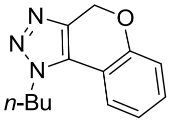

The general procedure was followed using NaN_3 (0.068 g, 1.05 mmol), CuI (19 mg, 0.100 mmol, 10 mol %), 1-iodo-2-(prop-2-yn-1-yloxy)benzene (**1a**, 0.256 g, 0.992 mmol), *n*-butyl bromide (0.144 g, 1.05 mmol) and LiO*t*-Bu (0.160 g, 2.00 mmol). Purification on silica gel (*n*-hexane/EtOAc = 10/1 → 5/1) gave **4c** (0.173 g, 76%) as a yellow oil. ^{1}H NMR (300 MHz, $CDCl_3$) δ 7.42 (dd, J = 8.0, 1.8 Hz, 1H), 7.30–7.23 (m, 1H), 7.08–7.01 (m, 2H), 5.44 (s, 2H), 4.59 (t, J = 7.4 Hz, 2H), 1.99–1.87 (m, 2H), 1.52–1.36 (m, 2H), 0.98 (t, J = 7.4 Hz, 3H); ^{13}C NMR (75 MHz, $CDCl_3$) δ 153.6 (C_q), 139.4 (C_q), 130.4 (CH), 126.9 (C_q), 122.1 (CH), 122.0 (CH), 118.1 (CH), 114.2 (C_q), 64.4 (CH_2), 49.6 (CH_2), 31.6 (CH_2), 19.7 (CH_2), 13.5 (CH_3); IR (film): 2961, 2872, 1619, 1559, 1520, 1460, 1336, 1201, 1124, 1022 cm^{-1}; MS (EI)

m/*z* (relative intensity) 229 (74) [M^+], 186 (12), 172 (24), 158 (100), 145 (40), 144 (34), 130 (16), 118 (28), 103 (32), 89 (24), 77 (26), 63 (12), 51 (8), 41 (28); HRMS (ESI) *m*/*z* calcd for $C_{13}H_{15}N_3O$ [$M+H^+$] 230.1288, found 230.1287. The spectral data were in accordance with those reported in the literature [3].

1-*n*-Hexyl-1,4-dihydrochromeno[4,3-*d*][1,2,3]triazole (4d):

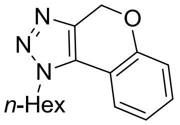

The general procedure was followed using NaN_3 (0.069 g, 1.06 mmol), CuI (19 mg, 0.100 mmol, 10 mol %), 1-iodo-2-(prop-2-yn-1-yloxy)benzene (**1a**, 0.258 g, 0.992 mmol), *n*-hexyl bromide (0.164 g, 0.993 mmol) and LiO*t*-Bu (0.163 g, 2.04 mmol). Purification on silica gel (*n*-hexane/EtOAc = 10/1 → 5/1) gave **4d** (0.150 g, 58%) as a yellow oil. ^{1}H NMR (300 MHz, $CDCl_3$) δ 7.40 (dd, *J* = 8.0, 1.6 Hz, 1H), 7.30–7.20 (m, 1H), 7.09–6.98 (m, 2H), 5.43 (s, 2H), 4.57 (t, *J* = 7.6 Hz, 2H), 2.01–1.86 (m, 2H), 1.47–1.20 (m, 6H), 0.87 (t, *J* = 7.0 Hz, 3H); ^{13}C NMR (75 MHz, $CDCl_3$) δ 153.6 (C_q), 139.4 (C_q), 130.4 (CH), 126.9 (C_q), 122.1 (CH), 122.0 (CH), 118.1 (CH), 114.2 (C_q), 64.5 (CH_2), 49.9 (CH_2), 31.1 (CH_2), 29.7 (CH_2), 26.1 (CH_2), 22.4 (CH_2), 13.9 (CH_3); IR (film): 2955, 2930, 1618, 1559, 1519, 1464, 1446, 1335, 1228, 1124, 1023 cm^{-1}; MS (EI) *m*/*z* (relative intensity): 257 (100) [M^+], 228 (10), 214 (22), 200 (18), 186 (38), 172 (36), 158 (78), 145 (52), 144 (26), 131 (16), 118 (20), 103 (24), 77 (18), 55 (14), 41 (30); HRMS (ESI) *m*/*z* calcd for $C_{15}H_{19}N_3O$ [$M+H^+$] 258.1601, found 258.1601. The spectral data were in accordance with those reported in the literature [4].

1-*n*-Decyl-1,4-dihydrochromeno[4,3-*d*][1,2,3]triazole (4e):

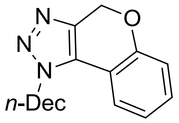

The general procedure was followed using NaN_3 (0.068 g, 1.05 mmol), CuI (19 mg, 0.100 mmol, 10 mol %), 1-iodo-2-(prop-2-yn-1-yloxy)benzene (**1a**, 0.259 g, 1.00 mmol), *n*-decyl bromide (0.224 g, 1.01 mmol) and LiO*t*-Bu (0.160 g, 2.00 mmol). Purification on silica gel (*n*-hexane/EtOAc = 10/1 → 5/1) gave **4e** (0.268 g, 86%) as a yellow oil. ^{1}H NMR (300 MHz, $CDCl_3$) δ 7.35 (dd, *J* = 8.0, 1.5 Hz, 1H), 7.25–7.15 (m, 1H), 7.02–6.93 (m, 2H), 5.38 (s, 2H), 4.57 (t, *J* = 7.5 Hz, 2H), 1.95–1.81 (m, 2H), 1.40–1.08 (m, 14H), 0.80 (t, *J* = 6.7 Hz, 3H); ^{13}C NMR (75 MHz, $CDCl_3$) δ 153.6 (C_q), 139.4 (C_q), 130.4 (CH), 126.9 (C_q), 122.1 (CH), 122.0 (CH), 118.1 (CH), 114.2 (C_q), 64.5 (CH_2), 49.9 (CH_2), 31.8 (CH_2), 29.7 (CH_2), 29.4 (CH_2), 29.3 (CH_2), 29.2 (CH_2), 29.0 (CH_2), 26.4 (CH_2), 22.6 (CH_2), 14.1 (CH_3); IR (film): 2927, 2846, 1519, 1462, 1373, 1336, 1194, 1119, 1022 cm^{-1}; MS (EI) *m*/*z* (relative intensity): 313 (36) [M^+], 214 (38), 200 (26), 186 (96), 173 (48), 158 (100), 145 (82), 118 (20), 103 (24), 77 (22), 55 (60), 41 (86); HRMS (ESI) *m*/*z* calcd for $C_{19}H_{27}N_3O$ [$M+H^+$] 314.2227, found 314.2227.

1-*n*-Undecyl-1,4-dihydrochromeno[4,3-*d*][1,2,3]triazole (4f):

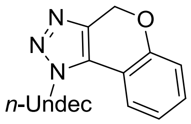

The general procedure was followed using NaN_3 (0.068 g, 1.05 mmol), CuI (19 mg, 0.100 mmol, 10 mol %), 1-iodo-2-(prop-2-yn-1-yloxy)benzene (**1a**, 0.259 g, 1.00 mmol), *n*-undecyl bromide (0.234 g, 0.995 mmol) and LiO*t*-Bu (0.160 g, 2.00 mmol). Purification on silica gel (*n*-hexane/EtOAc = 10/1 → 5/1) gave **4f** (0.240 g, 73%) as a brown solid. Mp = 40 °C; ^{1}H NMR (300 MHz, $CDCl_3$) δ 7.42 (dd,

J = 8.0, 1.6 Hz, 1H), 7.31–7.23 (m, 1H), 7.09–7.01 (m, 2H), 5.45 (s, 2H), 4.60 (t, J = 7.6 Hz, 2H), 2.02–1.88 (m, 2H), 1.48–1.02 (m, 16H), 0.87 (t, J = 6.7 Hz, 3H); ^{13}C NMR (75 MHz, $CDCl_3$) δ 153.7 (C_q), 139.4 (C_q), 130.4 (CH), 126.9 (C_q), 122.1 (CH), 122.0 (CH), 118.1 (CH), 114.3 (C_q), 64.5 (CH_2), 49.9 (CH_2), 31.9 (CH_2), 29.7 (CH_2), 29.5 (CH_2), 29.5 (CH_2), 29.4 (CH_2), 29.3 (CH_2), 29.0 (CH_2), 26.5 (CH_2), 22.6 (CH_2), 14.1 (CH_3); IR (KBr): 3051, 1618, 1559, 1520, 1464, 1447, 1404, 1376, 1264, 1201, 1124, 1039, 992, 842 cm^{-1}; MS (EI) m/z (relative intensity) 327 (100) [M^+], 311 (6), 228 (8), 220 (14), 214 (28), 186 (44), 158 (30), 145 (24), 103 (4), 43 (6); HRMS (ESI) m/z calcd for $C_{20}H_{29}N_3O$ [$M+H^+$] 328.2383, found 328.2383. The spectral data were in accordance with those reported in the literature [3].

1-(Cyclopropylmethyl)-1,4-dihydrochromeno[3,4-d][1,2,3]triazole (4g):

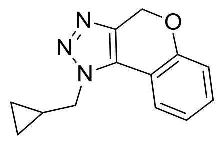

The general procedure was followed using NaN_3 (0.068 g, 1.05 mmol), CuI (19 mg, 0.100 mmol, 10 mol %), 1-iodo-2-(prop-2-yn-1-yloxy)benzene (**1a**, 0.259 g, 1.00 mmol), (bromomethyl)cyclopropane (0.136 g, 1.101 mmol) and LiO*t*-Bu (0.161 g, 2.01 mmol). Purification on silica gel (*n*-hexane/EtOAc = 10/1 → 5/1) gave **4g** (0.034 g, 15%) as a red oil. ^{1}H NMR (300 MHz, $CDCl_3$) δ 7.58–7.49 (m, 1H), 7.33–7.22 (m, 1H), 7.11–7.00 (m, 2H), 5.46 (s, 2H), 4.49 (d, J = 7.0 Hz, 2H), 1.48–1.31 (m, 1H), 0.71–0.57 (m, 2H), 0.55–0.46 (m, 2H); ^{13}C NMR (75 MHz, $CDCl_3$) δ 153.6 (C_q), 139.3 (C_q), 130.4 (CH), 127.0 (C_q), 122.1 (CH), 122.1 (CH), 118.1 (CH), 114.2 (C_q), 64.5 (CH_2), 54.0 (CH_2), 10.9 (CH), 4.1 (CH_2), 4.1 (CH_2); IR (film): 2940, 1689, 1532, 1445, 1401, 1211, 1058, 1001, 851 cm^{-1}; MS (EI) m/z (relative intensity): 227 (100) [M^+], 198 (14), 186 (6), 170 (16), 158 (48), 144 (24), 116 (12), 89 (14), 77 (12), 55 (74); HRMS (ESI) m/z calcd for $C_{13}H_{13}N_3O$ [$M+Na^+$] 250.0951, found 250.0957.

General procedure for the one-pot synthesis of triazoles 4

NaN_3 (1.05 equiv), CuI (10 mol %), LiO*t*-Bu (2.00 equiv), alkyne **1** (1.00 equiv) and alkyl bromide **2** (1.00 equiv) were dissolved in DMF (3.0 mL) and stirred at 80 °C for 20 h. Then, H_2O (50 mL) was added at ambient temperature, and the resulting mixture was extracted with EtOAc (3 × 50 mL). The combined organic layers were washed with saturated aq NH_4Cl (50 mL), H_2O (50 mL) and brine (50 mL), dried over Na_2SO_4, filtered and concentrated in vacuo. The remaining residue was purified by column chromatography on silica gel (*n*-hexane/EtOAc).

1-*n*-Octyl-1,4-dihydrochromeno[4,3-*d*][1,2,3]triazole (4a):

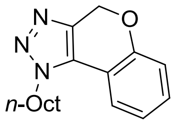

The general procedure was followed using NaN_3 (0.068 g, 1.05 mmol), CuI (19 mg, 0.100 mmol, 10 mol %), LiO*t*-Bu (0.160 g, 2.00 mmol), 1-iodo-2-(prop-2-yn-1-yloxy)benzene (**1a**, 0.263 g, 1.02 mmol), *n*-octyl bromide (0.197 g, 1.02 mmol). Purification on silica gel (*n*-hexane/EtOAc = 10/1 → 5/1) gave **4a** (0.1947 g, 68%) as a yellow oil.

1-*n*-Butyl-1,4-dihydrochromeno[4,3-*d*][1,2,3]triazole (4c):

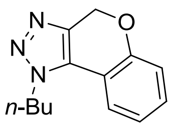

The general procedure was followed using NaN_3 (0.068 g, 1.05 mmol), CuI (19 mg, 0.100 mmol, 10 mol %), LiO*t*-Bu (0.160 g, 2.00 mmol), 1-iodo-2-(prop-2-yn-1-yloxy)benzene (**1a**, 0.258 g, 1.00 mmol), *n*-butyl bromide (0.138 g, 1.01 mmol). Purification on silica gel (*n*-hexane/EtOAc = 10/1 → 5/1) gave **4c** (0.172 g, 75%) as a yellow oil.

1-*n*-Hexyl-1,4-dihydrochromeno[4,3-*d*][1,2,3]triazole (4d):

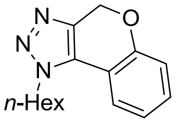

The general procedure was followed using NaN_3 (0.068 g, 1.05 mmol), CuI (19 mg, 0.100 mmol, 10 mol %), LiO*t*-Bu (0.160 g, 2.00 mmol), 1-iodo-2-(prop-2-yn-1-yloxy)benzene (**1a**, 0.258 g, 1.00 mmol), and *n*-hexyl bromide (0.168 g, 1.02 mmol). Purification on silica gel (*n*-hexane/EtOAc = 10/1 → 5/1) gave **4d** (0.184 g, 72%) as a yellow oil.

1-*n*-Undecyl-1,4-dihydrochromeno[4,3-*d*][1,2,3]triazole (4f):

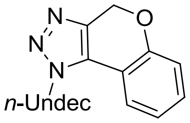

The general procedure was followed using NaN_3 (0.068 g, 1.05 mmol), CuI (19 mg, 0.100 mmol, 10 mol %), 1-iodo-2-(prop-2-yn-1-yloxy)benzene (**1a**, 0.259 g, 1.00 mmol), *n*-undecyl bromide (0.234 g, 0.995 mmol) and LiO*t*-Bu (0.160 g, 2.00 mmol). Purification on silica gel (*n*-hexane/EtOAc = 10/1 → 5/1) gave **4f** (0.240 g, 78%) as a brown solid.

1-*n*-Octyl-4,6-dihydro-1*H*-benzo[5,6]oxepino[3,4-d][1,2,3]triazole (4h):

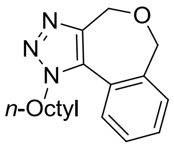

The general procedure was followed using NaN_3 (0.068 g, 1.05 mmol), CuI (19 mg, 0.100 mmol, 10 mol %), 1-iodo-2-[(prop-2-yn-1-yloxy)methyl]benzene (0.272 g, 1.00 mmol), *n*-octyl bromide (0.195 g, 1.01 mmol) and LiO*t*-Bu (0.160 g, 2.00 mmol). Purification on silica gel (*n*-hexane/EtOAc = 10/1 → 5/1 → 2/1) gave **4h** (0.220 g, 75%) as a yellow oil. ^{1}H NMR (300 MHz, $CDCl_3$) δ 7.58 (d, *J* = 7.6 Hz, 1H), 7.53–7.44 (m, 1H), 7.43–7.37 (m, 2H), 5.14 (s, 2H), 4.60–4.49 (m, 4H), 2.03–1.88 (m, 2H), 1.41–1.12 (m, 10H), 0.86 (t, *J* = 6.8 Hz, 3H); ^{13}C NMR (75 MHz, $CDCl_3$) δ 144.6 (C_q), 139.5 (C_q), 131.7 (C_q), 129.9 (CH), 128.8 (CH), 128.6 (CH), 126.5 (CH), 126.4 (C_q), 70.4 (CH_2), 67.1 (CH_2), 49.5 (CH_2), 31.6 (CH_2), 30.0 (CH_2), 29.0 (CH_2), 28.9 (CH_2), 26.5 (CH_2), 22.6 (CH_2), 14.0 (CH_3); IR (film): 2987, 2857, 1624, 1598, 1536, 1460, 1209, 1128, 1018 cm^{-1}; MS (EI) *m*/*z* (relative intensity): 300 (60) [M^+], 284 (8), 271 (8), 257 (8), 242 (16), 228 (8), 214 (12), 200 (74) 187 (42), 172 (10), 159 (100), 130 (34), 115 (40), 103 (26), 89 (8), 77 (12), 57 (26), 43 (56), 41 (52); HRMS (ESI) *m*/*z* calcd for $C_{18}H_{25}N_3O$ [$M+H^+$] 300.2070, found 300.2072.

1-*n*-Butyl-4,6-dihydro-1*H*-benzo[5,6]oxepino[3,4-d][1,2,3]triazole (4i):

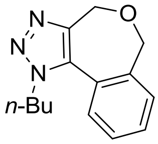

The general procedure was followed using NaN_3 (0.068 g, 1.05 mmol), CuI (19 mg, 0.100 mmol, 10 mol %), 1-iodo-2-[(prop-2-yn-1-yloxy)methyl]benzene (0.272 g, 1.00 mmol), *n*-butyl bromide (0.139 g, 1.01 mmol) and LiO*t*-Bu (0.160 g, 2.00 mmol). Purification on silica gel (*n*-hexane/EtOAc = 10/1 → 5/1 → 2/1) gave **4i** (0.123 g,

51%) as yellow oil. ^{1}H NMR (300 MHz, $CDCl_3$) δ 7.59 (d, *J* = 7.7 Hz, 1H), 7.53–7.44 (m, 1H), 7.42–7.37 (m, 2H), 5.13 (s, 2H), 4.59–4.51 (m, 4H), 2.02–1.88 (m, 2H), 1.47–1.31 (m, 2H), 0.94 (t, *J* = 7.4 Hz, 3H); ^{13}C NMR (75 MHz, $CDCl_3$) δ 144.6 (C_q), 139.5 (C_q), 131.7 (C_q), 129.9 (CH), 128.8 (CH), 128.6 (CH), 126.5 (CH), 126.4 (C_q), 70.4 (CH_2), 67.1 (CH_2), 49.3 (CH_2), 32.1 (CH_2), 19.8 (CH_2), 13.5 (CH_3); IR (film): 2954, 2930, 1625, 1557, 1481, 1457, 1326, 1225, 1136, 1022 cm^{-1}; MS (EI) *m*/*z* (relative intensity): 243 (100) [M^+], 201 (64), 172 (6), 159 (96), 144 (12), 130 (60), 115 (54), 103 (44) 89 (16), 77 (22), 57 (18), 41 (42); HRMS (ESI) *m*/*z* calcd for $C_{14}H_{17}N_3O$ [$M+Na^+$] 266.1264, found 266.1271.

1-*n*-Hexyl-4,6-dihydro-1*H*-benzo[5,6]oxepino[3,4-d][1,2,3]triazole (4j):

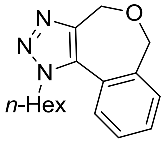

The general procedure was followed using NaN_3 (0.068 g, 1.05 mmol), CuI (19 mg, 0.100 mmol, 10 mol %), 1-iodo-2-[(prop-2-yn-1-yloxy)methyl]benzene (0.272 g, 1.00 mmol), *n*-hexyl bromide (0.165 g, 1.00 mmol) and LiO*t*-Bu (0.160 g, 2.00 mmol). Purification on silica gel (*n*-hexane/EtOAc = 10/1 → 5/1 → 3/1) gave **4j** (0.168 g, 62%) as yellow oil. ^{1}H NMR (300 MHz, $CDCl_3$) δ 7.58 (d, *J* = 7.6 Hz, 1H), 7.54–7.44 (m, 1H), 7.42–7.37 (m, 2H), 5.14 (s, 2H), 4.60–4.50 (m, 4H), 2.03–1.90 (m, 2H), 1.41–1.21 (m, 6H), 0.86 (t, *J* = 7.0 Hz, 3H); ^{13}C NMR (75 MHz, $CDCl_3$) δ 144.6 (C_q), 139.5 (C_q), 131.7 (C_q), 129.9 (CH), 128.8 (CH), 128.6 (CH), 126.5 (CH), 126.4 (C_q), 70.4 (CH_2), 67.1 (CH_2), 49.3 (CH_2), 32.1 (CH_2), 30.0 (CH_2), 26.2 (CH_2), 22.4 (CH_2), 13.9 (CH_3); IR (film): 2950, 2930, 1624, 1555, 1519, 1450, 1330, 1228, 1129, 1022 cm^{-1}; MS (EI) *m*/*z* (relative intensity): 271 (52) [M^+], 255 (6), 243 (24), 201 (84), 187 (14), 172 (10), 159 (100), 144 (12), 130 (42), 115 (36), 103 (26) 89 (8), 77 (12), 55

(16), 43 (56), 41 (28); HRMS (ESI) *m*/*z* calcd for $C_{16}H_{21}N_3O$ [M+H$^+$] 272.1757, found 272.1757.

4-Methyl-1-*n*-octyl-1,4-dihydrochromeno[4,3-*d*][1,2,3]-triazole (4k):

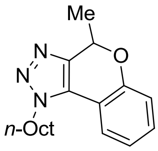

The general procedure was followed using NaN_3 (0.068 g, 1.05 mmol), CuI (19 mg, 0.100 mmol, 10 mol %), 1-(but-3-yn-2-yloxy)-2-iodobenzene (0.272 g, 1.00 mmol), *n*-octyl bromide (0.195 g, 1.01 mmol) and LiO*t*-Bu (0.160 g, 2.00 mmol). Purification on silica gel (*n*-hexane/EtOAc = 1/0 → 20/1 → 10/1 →5/1) gave **4k** (0.089 g, 30%) as a yellow oil. ^{1}H NMR (300 MHz, $CDCl_3$) δ 7.41 (dd, *J* = 8.0, 1.5 Hz, 1H), 7.30–7.21 (m, 1H), 7.08–7.00 (m, 2H), 5.61 (q, *J* = 6.5 Hz, 1H), 4.65–4.47 (m, 2H), 2.02–1.87 (m, 2H), 1.76 (d, *J* = 6.5 Hz, 3H), 1.49–1.14 (m, 10H), 0.86 (t, *J* = 6.8 Hz, 3H); ^{13}C NMR (75 MHz, $CDCl_3$) δ 153.3 (C_q), 143.5 (C_q), 130.3 (CH), 126.3 (C_q), 121.9 (CH), 121.9 (CH), 118.3 (CH), 114.1 (C_q), 71.6 (CH), 49.9 (CH_2), 31.7 (CH_2), 29.7 (CH_2), 29.0 (CH_2), 29.0 (CH_2), 26.5 (CH_2), 22.6 (CH_2), 20.0 (CH_3), 14.0 (CH_3); IR (film): 2901, 2859, 1499, 1478, 1402, 1347, 1225, 1134, 1027 cm^{-1}; MS (EI) *m*/*z* (relative intensity): 299 (86) [M$^+$], 284 (46), 270 (4), 256 (32), 242 (6), 228 (22), 220 (24), 186 (42), 172 (100), 159 (42), 131 (10), 115 (14), 103 (6), 55 (18), 43 (30), 41 (36); HRMS (ESI) *m*/*z* calcd for $C_{18}H_{25}N_3O$ [M+H$^+$] 300.2070, found 300.2075.

1-*n*-Octyl-4,5-dihydro-1*H*-naphtho[1,2-*d*][1,2,3]-triazole (4l):

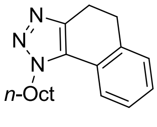

The general procedure was followed using NaN_3 (0.068 g, 1.05 mmol), CuI (19 mg, 0.100 mmol, 10 mol %), 1-iodo-2-(prop-2-yn-1-yl)benzene (0.255 g, 0.996 mmol),

n-octyl bromide (0.1921 g, 0.994 mmol) and LiO*t*-Bu (0.160 g, 2.00 mmol). Purification on silica gel (*n*-hexane/EtOAc = 10/1 → 5/1 → 3/1) gave **4l** (0.202 g, 70%) as a brown oil. ^{1}H NMR (300 MHz, $CDCl_3$) δ 7.46 (d, *J* = 7.7 Hz, 1H), 7.36–7.19 (m, 3H), 4.59 (t, *J* = 7.5 Hz, 2H), 3.09–2.89 (m, 4H), 2.01–1.86 (m, 2H), 1.48–1.10 (m, 10H), 0.84 (t, *J* = 6.7 Hz, 3H); ^{13}C NMR (75 MHz, $CDCl_3$) δ 145.4 (C_q), 137.2 (C_q), 130.7 (C_q), 129.3 (CH), 128.3 (CH), 127.1 (CH), 125.4 (C_q), 121.9 (CH), 50.0 (CH_2), 31.7 (CH_2), 30.3 (CH_2), 29.7 (CH_2), 29.0 (CH_2), 29.0 (CH_2), 26.5 (CH_2), 22.6 (CH_2), 20.7 (CH_2), 14.0 (CH_3); IR (film): 3056, 2364, 1712, 1509, 1374, 1306, 1196, 1085, 1013, 723 cm^{-1}; MS (EI) *m*/*z* (relative intensity): 283 (42) [M^+], 226 (16), 184 (76), 171 (32), 156 (16), 143 (100), 128 (14), 115 (22), 43 (8); HRMS (ESI) *m*/*z* calcd for $C_{18}H_{25}N_3$ [$M+Na^+$] 306.1941, found 306.1938. The spectral data were in accordance with those reported in the literature [3].

5-Methyl-1-*n*-octyl-4,5-dihydro-1*H*-[1,2,3]triazolo[4,5-*c*]quinoline (4m):

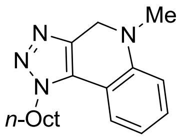

The general procedure was followed using NaN_3 (0.068 g, 1.05 mmol), CuI (19 mg, 0.100 mmol, 10 mol %), 2-iodo-*N*-methyl-*N*-(prop-2-yn-1-yl)aniline (0.271 g, 1.00 mmol), *n*-octyl bromide (0.193 g, 1.00 mmol) and LiO*t*-Bu (0.160 g, 2.00 mmol). Purification on silica gel (*n*-hexane/EtOAc = 10/1 → 4/1 → 2/1) gave **4m** (0.193 g, 65%) as a brown oil. ^{1}H NMR (300 MHz, $CDCl_3$) δ 7.37–7.32 (m, 1H), 7.26–7.19 (m, 1H), 6.83–6.76 (m, 1H), 6.73–6.68 (d, *J* = 8.4 Hz, 1H), 4.63 (s, 2H), 4.58 (t, *J* = 7.7 Hz, 2H), 2.94 (s, 3H), 2.01–1.86 (m, 2H), 1.48–1.18 (m, 10H), 0.91–0.83 (m, 3H); ^{13}C NMR (75 MHz, $CDCl_3$) δ 145.5 (C_q), 140.1 (C_q), 130.0 (CH), 128.3 (C_q), 122.3 (CH), 117.6 (CH), 112.6 (C_q), 112.5 (CH), 50.0 (CH_2), 49.7 (CH_2), 49.7 (CH_2), 38.8 (CH_3), 31.7 (CH_2), 29.5 (CH_2), 29.0 (CH_2), 26.5 (CH_2), 22.6 (CH_2), 14.0 (CH_3);

IR (film): 2925, 2858, 1737, 1685, 1619, 1520, 1458, 1359, 1244, 1204, 1047 cm^{-1}; MS (EI) *m*/*z* (relative intensity) 297 (100) [M$^+$], 269 (5), 185 (5), 171 (10), 157 (30), 142 (10); HRMS (ESI) *m*/*z* calcd for $C_{18}H_{26}N_4$ [M+H$^+$] 299.2230, found 299.2232.

General procedure for the copper-catalyzed one-pot C–C/C–N arylation with azoles 5

Azole **5** (1.00 mmol), aryl iodide **6** (5.00 mmol), CuI (20 mol %) and LiO*t*-Bu (3.00 mmol) were dissolved in DMF (3.0 mL) and stirred at 140 ºC for 22 h under N_2. H_2O (75 mL) was added at ambient temperature, and the resulting mixture was extracted with MTBE (3 × 75 mL). The combined organic layers were washed with H_2O (50 mL) and brine (50 mL), dried over Na_2SO_4 and concentrated in vacuo. The residue was purified by column chromatography on silica gel (*n*-hexane/EtOAc).

1,2-Diphenyl-1*H*-benzo[*d*]imidazole (7a):

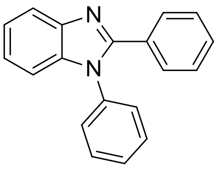

The general procedure was followed using benzimidazole (0.118 g, 1.00 mmol), iodobenzene (1.02 g, 5.00 mmol), CuI (38 mg, 0.20 mmol, 20 mol %) and LiO*t*-Bu (0.240 g, 3.00 mmol). Purification on silica gel (*n*-hexane/EtOAc: 5/1) yielded **7a** (208 mg, 77%) as a colorless solid. Mp = 110 °C; ^{1}H NMR (300 MHz, $CDCl_3$) δ 7.88 (m, 1H), 7.58–7.54 (m, 2H), 7.52–7.44 (m, 3H), 7.36–7.22 (m, 8H); ^{13}C NMR (75 MHz, $CDCl_3$) δ 152.3 (C_q), 142.9 (C_q), 137.1 (C_q), 136.9 (C_q), 129.9 (C_q), 129.8 (CH), 129.4 (CH), 129.4 (CH), 128.5 (CH), 128.2 (CH), 127.4 (CH), 123.3 (CH), 122.9 (CH), 119.8 (CH), 110.4 (CH); IR (KBr): 3525, 2333, 1596, 1492, 1477, 1456, 1444, 1328, 1260, 1181, 1077, 976, 832, 764, 751, 707 cm^{-1}; MS (EI) *m*/*z* (relative intensity) 270 (88) [M$^+$], 269 (100), 166 (5), 77 (12), 51 (7); HRMS (ESI) *m*/*z* calcd for

$C_{19}H_{14}N_2$ [M+H$^+$] 271.1229, found 271.1229. The spectral data were in accordance with those reported in the literature [5].

1,2-Di-*m*-tolyl-1*H*-benzo[*d*]imidazole (7b):

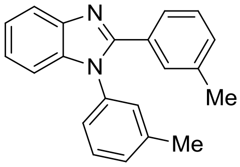

The general procedure was followed using benzimidazole (0.118 g, 1.00 mmol), 3-iodotoluene (1.10 g, 5.00 mmol), CuI (38 mg, 0.20 mmol, 20 mol %) and LiO*t*-Bu (0.240 g, 3.00 mmol). Purification on silica gel (n-hexane/EtOAc: 9/1 → 5/1 → 0/1) yielded **7b** (243 mg, 82%) as an off-white solid. Mp = 103–105 °C; ^{1}H NMR (300 MHz, $CDCl_3$) δ 7.87 (dt, *J* = 7.8, 0.8, Hz, 1H), 7.61 (m, 1H), 7.38–7.03 (m, 10H), 2.37 (s, 3H), 2.30 (s, 3H); ^{13}C NMR (75 MHz, $CDCl_3$) δ 152.4 (C_q), 142.9 (C_q), 139.8 (C_q), 138.0 (C_q), 137.2 (C_q), 136.9 (C_q), 130.1 (CH), 130.1 (CH), 129.8 (C_q), 129.5 (CH), 129.2 (CH), 127.8 (CH), 127.7 (CH), 126.2 (CH), 124.5 (CH), 123.1 (CH), 122.7 (CH), 119.6 (CH), 110.4 (CH), 21.3 (CH_3), 21.3 (CH_3); IR (KBr): 3779, 2521, 1599, 1490, 1458, 1377, 1326, 1267, 1089, 882, 802, 751, 706 cm^{-1}; MS (EI) *m*/*z* (relative intensity) 298 (100) [M$^+$], 297 (88), 282 (8), 180 (4), 148 (2), 91 (4), 65 (6); HRMS (ESI) *m*/*z* calcd for $C_{21}H_{18}N_2$ [M+H$^+$] 299.1543, found 299.1543.

1,2-Di-*p*-tolyl-1*H*-benzo[*d*]imidazole (7c):

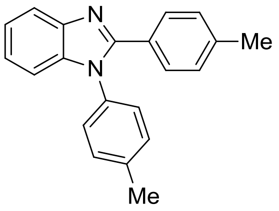

The general procedure was followed using benzimidazole (0.118 g, 1.00 mmol), 4-iodotoluene (1.09 g, 5.00 mmol), CuI (38 mg, 0.20 mmol, 20 mol %) and LiO*t*-Bu (0.240 g, 3.00 mmol). Purification on silica gel (*n*-hexane/EtOAc: 9/1 → 5/1) yielded **7c** (215 mg, 72%) as an off-white solid. Mp = 108 °C; ^{1}H NMR (300 MHz, $CDCl_3$) δ 7.87 (dm, *J* = 8.0 Hz, 1H), 7.48 (d, *J* = 8.4 Hz, 2H), 7.34–7.10 (m, 9H), 2.45 (s, 3H), 2.34 (s, 3H); ^{13}C NMR (75 MHz, $CDCl_3$) δ 152.5 (C_q), 142.9 (C_q), 139.4 (C_q), 138.4 (C_q), 137.3 (C_q), 134.4 (C_q), 130.4 (CH), 129.3 (CH), 129.0 (CH), 127.2 (CH), 127.1 (C_q), 123.0 (CH), 122.7 (CH), 119.6 (CH), 110.4 (CH), 21.3 (CH_3), 21.2 (CH_3); IR (KBr): 3638, 2531, 1609, 1515, 1480, 1453, 1382, 1262, 1185, 1020, 848, 747 cm^{-1}; MS (EI) *m*/*z* (relative intensity) 298 (100) [M$^+$], 297 (86), 282 (4), 180 (6), 149 (3), 116 (3), 91 (6), 65 (8); HRMS (ESI) *m*/*z* calcd for $C_{21}H_{18}N_2$ [M+H$^+$] 299.1543, found 299.1543.

1,2-Bis(4-methoxyphenyl)-1*H*-benzo[*d*]imidazole (7d)

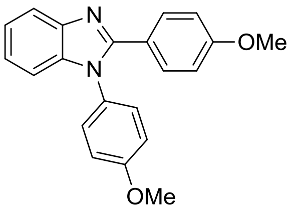

The general procedure was followed using benzimidazole (0.118 g, 1.00 mmol), 4-iodoanisole (1.17 g, 5.00 mmol), CuI (38 mg, 0.20 mmol, 20 mol %) and LiO*t*-Bu (0.240 g, 3.00 mmol). Purification on silica gel (n-hexane/EtOAc: 9/1 → 5/1 → 4/1) yielded **7d** (196 mg, 59%) as an off-white solid. Mp = 150–152 °C; ^{1}H NMR

(300 MHz, $CDCl_3$) δ 7.85 (d, *J* = 7.9 Hz, 1H), 7.54 (d, *J* = 8.9 Hz, 2H), 7.33–7.12 (m, 5H), 7.01 (d, *J* = 8.9 Hz, 2H), 6.83 (d, *J* = 8.9 Hz, 2H), 3.88 (s, 3H), 3.80 (s, 3H); ^{13}C NMR (75 MHz, $CDCl_3$) δ 160.4 (C_q), 159.4 (C_q), 152.4 (C_q), 142.8 (C_q), 137.5 (C_q), 130.8 (CH), 130.0 (C_q), 128.6 (CH), 122.8 (CH), 122.7 (CH), 122.4 (C_q), 119.4 (CH), 114.9 (CH), 113.7 (CH), 110.2 (CH), 55.5 (CH_3), 55.2 (CH_3); IR (KBr): 2995, 2838, 2544, 1610, 1455, 1426, 1249, 1181, 1106, 1031, 851, 751 cm^{-1}; MS (EI) *m*/*z* (relative intensity) 330 (100) [M^+], 286 (17), 243 (11), 128 (9), 77 (6); HRMS (ESI) *m*/*z* calcd for $C_{21}H_{18}N_2O_2$ [$M+H^+$] 330.1368, found 330.1377.

1,2-Bis(4-chlorophenyl)-1*H*-benzo[*d*]imidazole (7e)

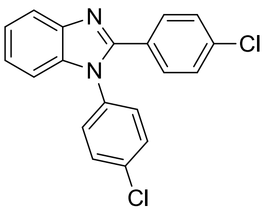

The general procedure was followed using benzimidazole (0.118 g, 1.00 mmol), 4-chloroiodobenzene (1.20 g, 5.00 mmol), CuI (38 mg, 0.20 mmol, 20 mol %) and LiO*t*-Bu (0.240 g, 3.00 mmol). Purification on silica gel (*n*-hexane/ EtOAc: 5/1) yielded **7e** (210 mg, 62%) as a colorless solid. Mp = 152–155 °C; ^1H NMR (300 MHz, $CDCl_3$) δ 7.88 (ddd, *J* = 8.0, 1.3, 0.8 Hz, 1H), 7.53–7.48 (m, 4H), 7.39–7.21 (m, 7H); ^{13}C NMR (75 MHz, $CDCl_3$) δ 151.1 (C_q), 142.9 (C_q), 136.9 (C_q), 135.9 (C_q), 135.2 (C_q), 134.6 (C_q), 130.6 (CH), 130.3 (CH), 128.8 (CH), 128.5 (CH), 128.1 (C_q), 123.8 (CH), 123.3 (CH), 120.0 (CH), 110.2 (CH); IR (KBr): 3547, 2497, 1595, 1495, 1451, 1409, 1378, 1262, 1199, 1090, 1015, 744 cm^{-1}; MS (EI) *m*/*z* (relative intensity) 338 (100) [M^+], 302(12), 166 (6), 111 (6), 75 (8); HRMS (EI) *m*/*z* calcd for $C_{19}H_{13}Cl_2N_2$ [$M+H^+$] 339.0450, found 339.0450.

1,2-Diphenyl-1*H*-imidazole (7f)

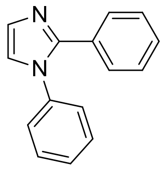

The general procedure was followed using imidazole (68 mg, 1.00 mmol), iodobenzene (1.02 g, 5.00 mmol), CuI (38 mg, 0.20 mmol, 20 mol %) and LiO*t*-Bu (0.240 g, 3.00 mmol). Purification on silica gel (*n*-hexane/EtOAc: 7/3) yielded **7f** (94 mg, 43%) as an off-white solid. Mp = 85–87 °C. ^{1}H NMR (300 MHz, $CDCl_3$) δ 7.40–7.36 (m, 5H), 7.29–7.14 (m, 7H); ^{13}C NMR (75 MHz, $CDCl_3$) δ 146.6 (C_q), 138.5 (C_q), 130.3 (C_q), 129.4 (CH), 129.0 (CH), 128.5 (CH), 128.2 (CH), 128.1 (CH), 128.0 (CH), 125.8 (CH), 122.8 (CH); IR (KBr): 3132, 1597, 1493, 1462, 1415, 1313, 1137, 970, 773, 691 cm^{-1}; MS (EI) *m*/*z* (relative intensity) 219 (100) [M-H^{+}], 193 (25), 178 (17), 117 (19), 90 (33), 77 (52), 51 (38); HRMS (ESI) *m*/*z* calcd for $C_{15}H_{12}N_2$ [M+H^{+}] 220.1000, found 220.0998. The spectral data were in accordance with those reported in the literature [6].

References

[1] Kacprzak, K. *Synlett* **2005**, 943–946.

[2] (a) Bowman, W. R.; Krintel, S. L.; Schilling, M. B. *Org. Biomol. Chem.* **2004**, *2*, 585-592. (b) Pastine, S. J.; Youn, S. W.; Sames, D. *Org. Lett.* **2003**, *5,* 1055-1058. (c) Wang, R. T.; Chou, F. L.; Luo, F. T. *J. Org. Chem.* **1990**, *55*, 4846-4849. (d) Shore, G.; Organ, M. G. *Chem. Eur. J.* **2008**, *14*, 9641-9646.

[3] Ackermann, L.; Jeyachandran, R.; Potukuchi, H. K.; Novák, P.; Büttner, L. *Org. Lett.* **2010**, *12*, 2056-2059.

[4] Schulman, J. M.; Friedman, A. A.; Panteleev, J.; Lautens, M. *Chem. Comm.* **2012**, *48*, 55-57.

[5] Saha, P.; Ramana, T.; Purkait, N.; Ali, M. A.; Paul, R.; Punniyamurthy, T. *J. Org. Chem.* **2009**, *74*, 8719–8725.

[6] Wentzel, M. T.; Hewgley, J. B.; Kamble, R. M.; Wall, P. D.; Kozlowski, M. C. *Adv. Synth. Catal.* **2009**, *351*, 931–937.

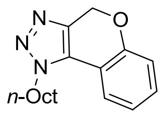

4a
(CDCl$_3$, 300 MHz)

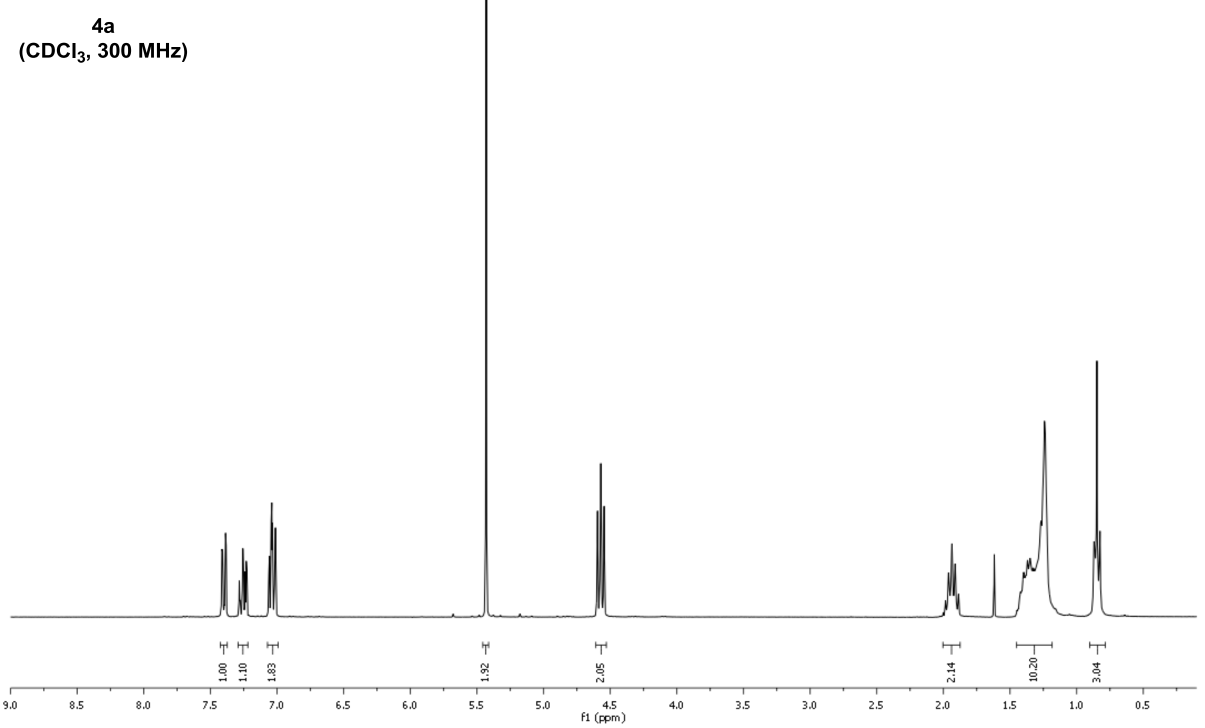

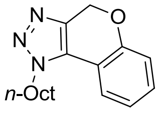

4a
(CDCl$_3$, 75 MHz)

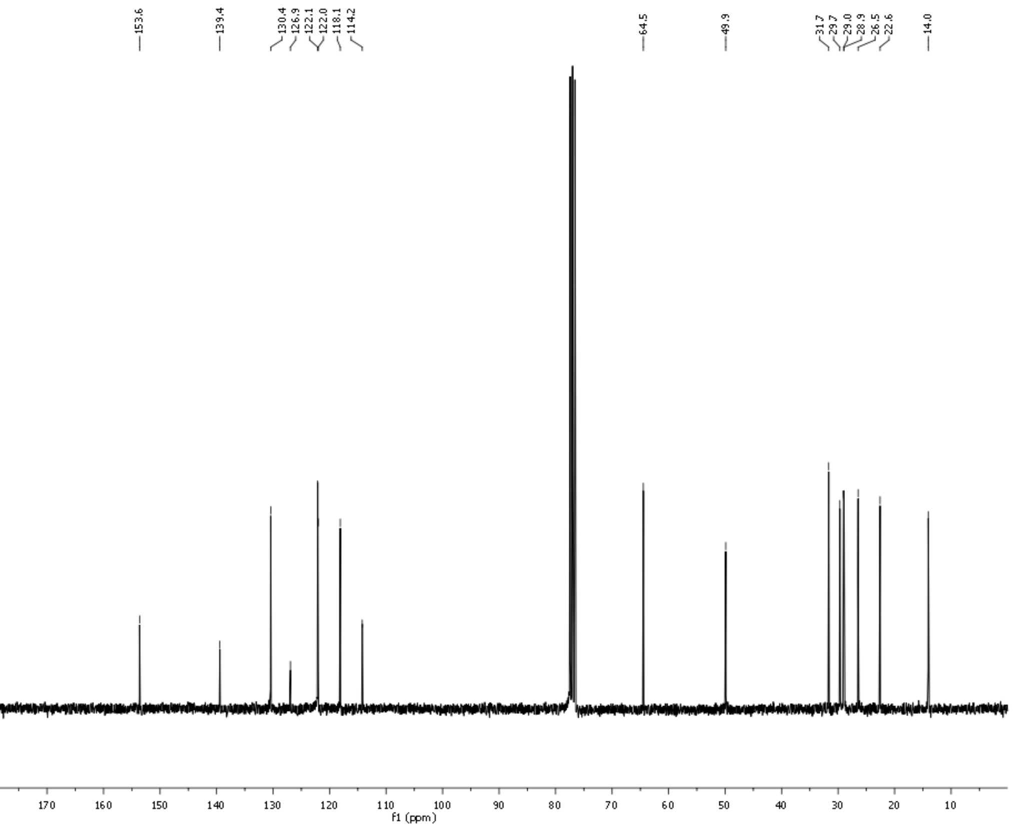

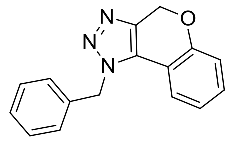

4b
($CDCl_3$, 300 MHz)

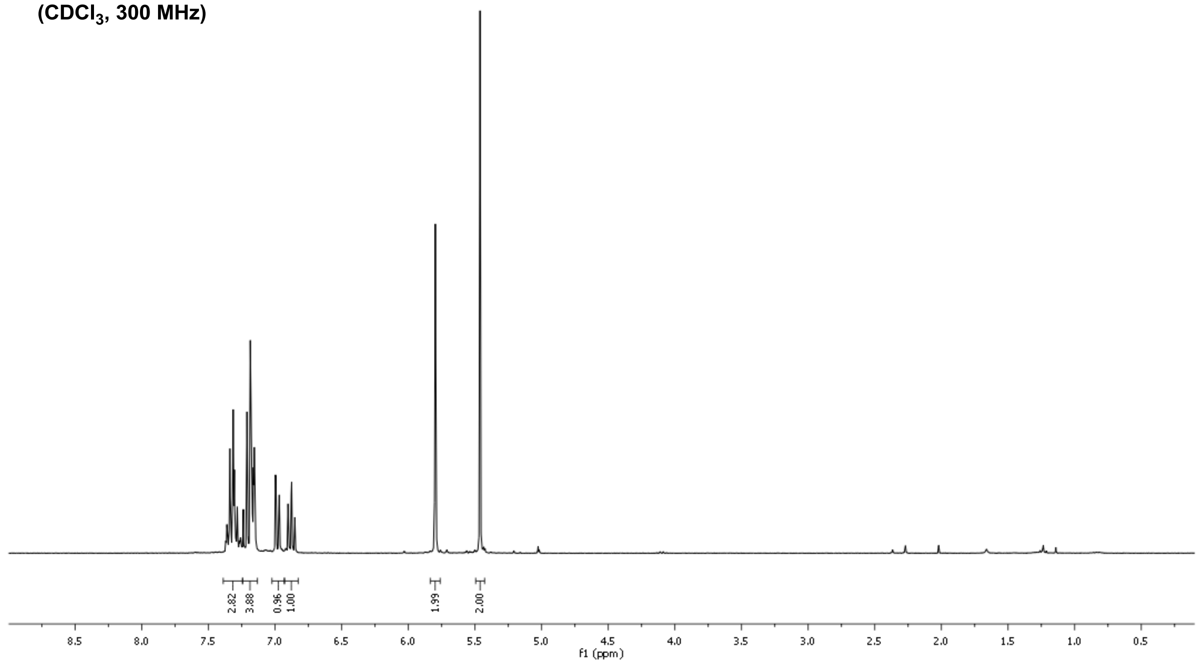

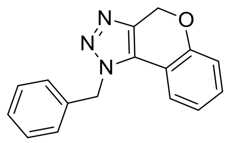

4b
($CDCl_3$, 75 MHz)

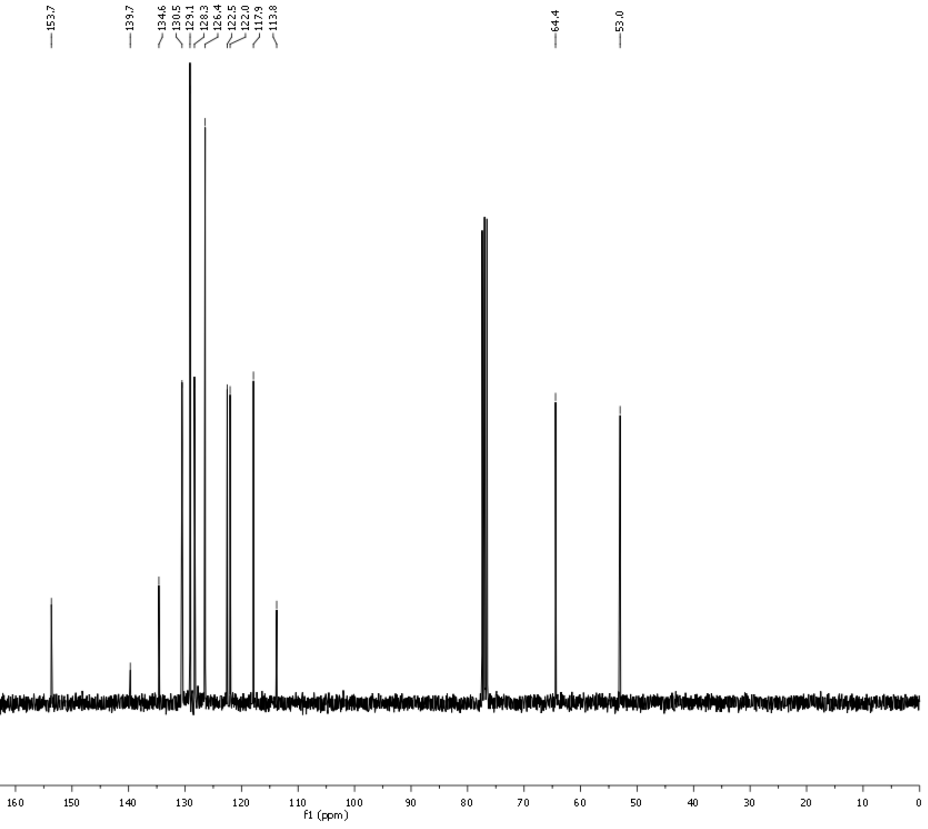

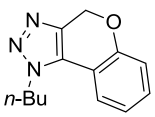

4c
($CDCl_3$, 300 MHz)

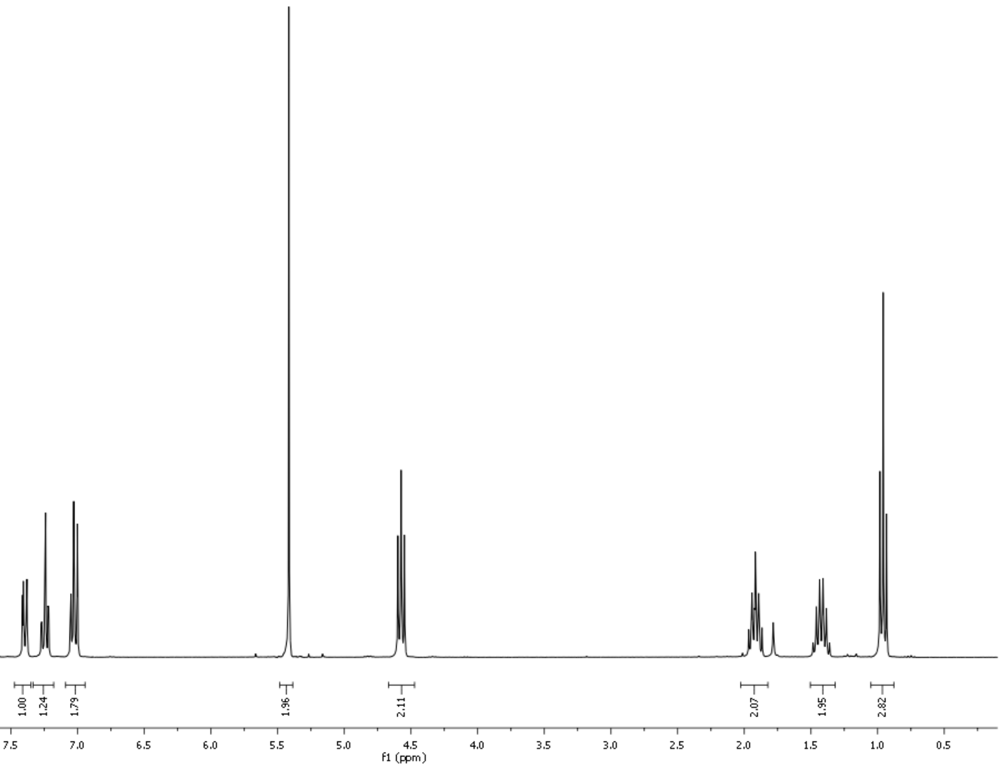

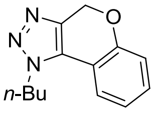

4c
($CDCl_3$, 75 MHz)

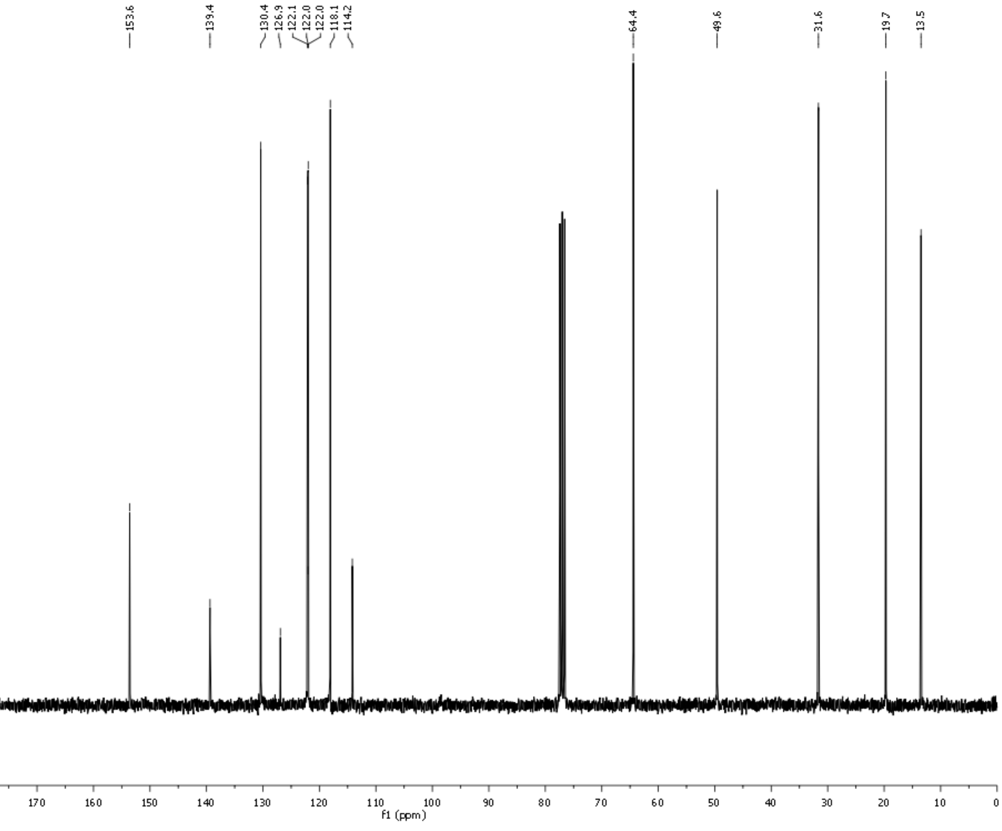

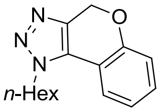

4d
($CDCl_3$, 300 MHz)

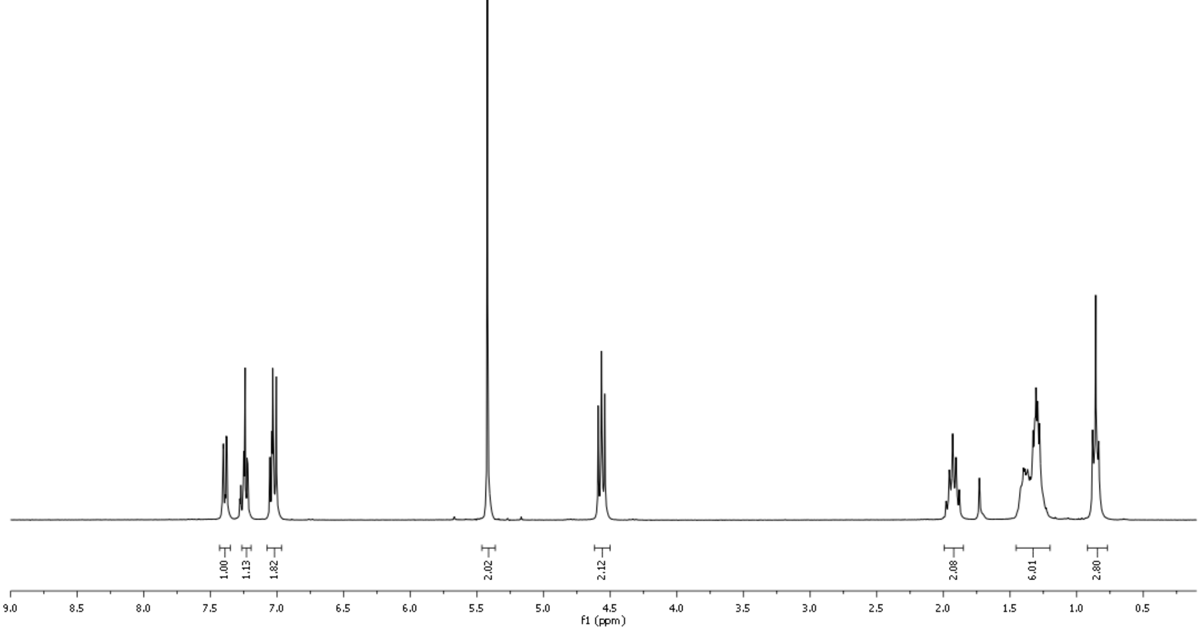

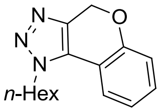

4d
($CDCl_3$, 75 MHz)

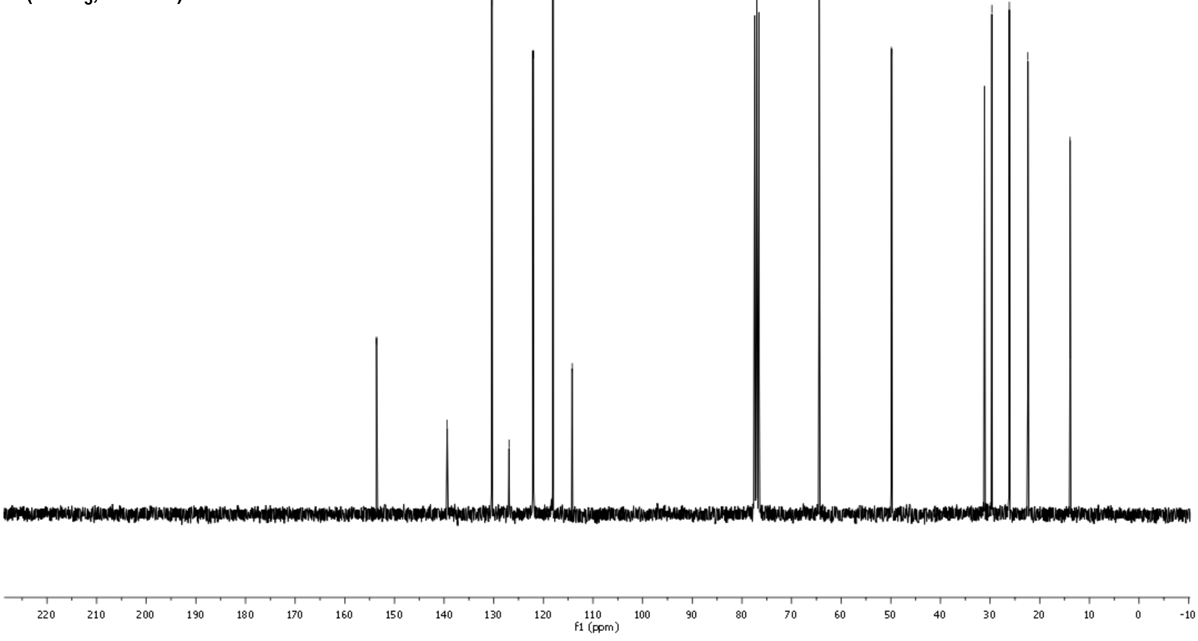

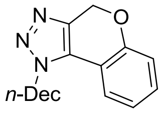

4e
($CDCl_3$, 300 MHz)

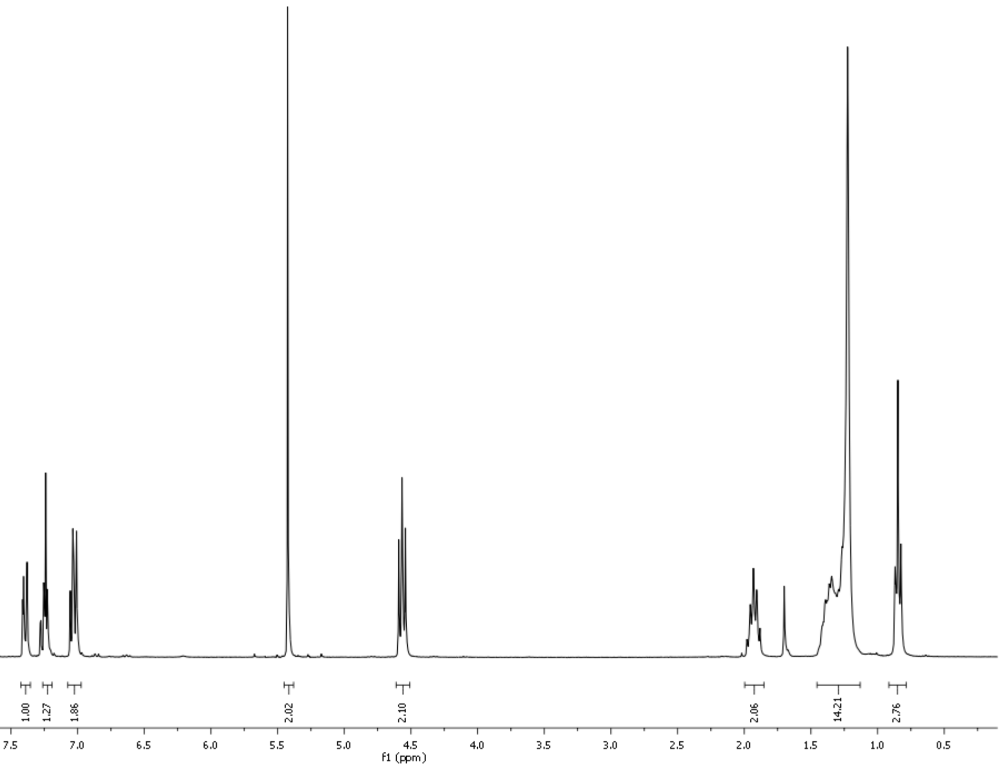

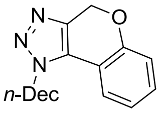

4e
($CDCl_3$, 75 MHz)

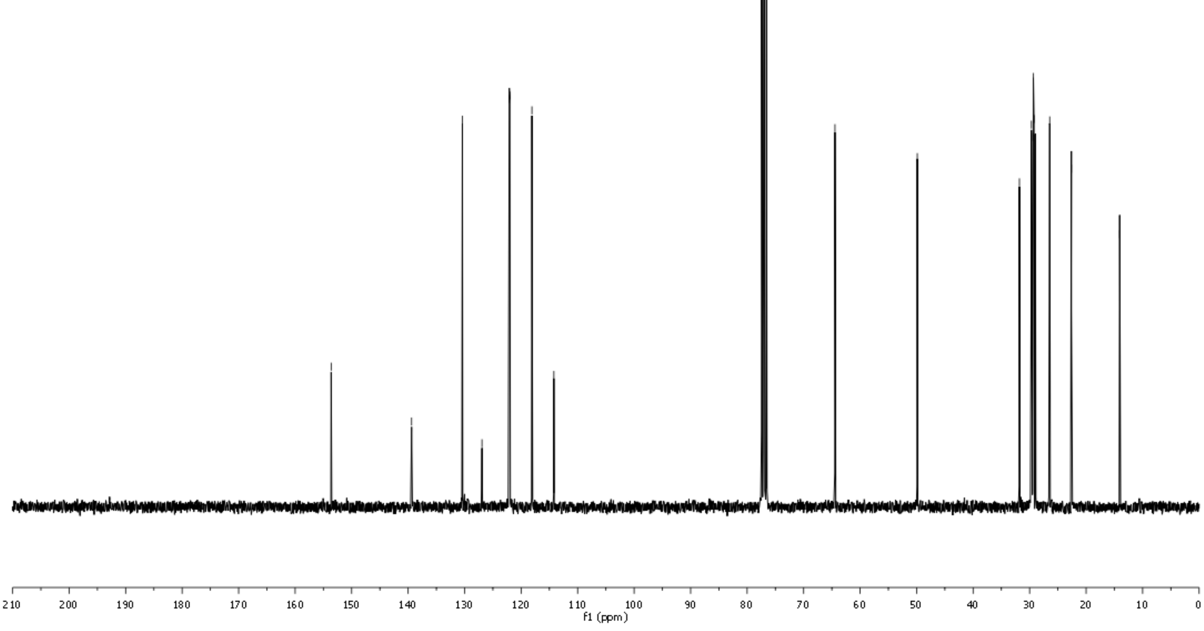

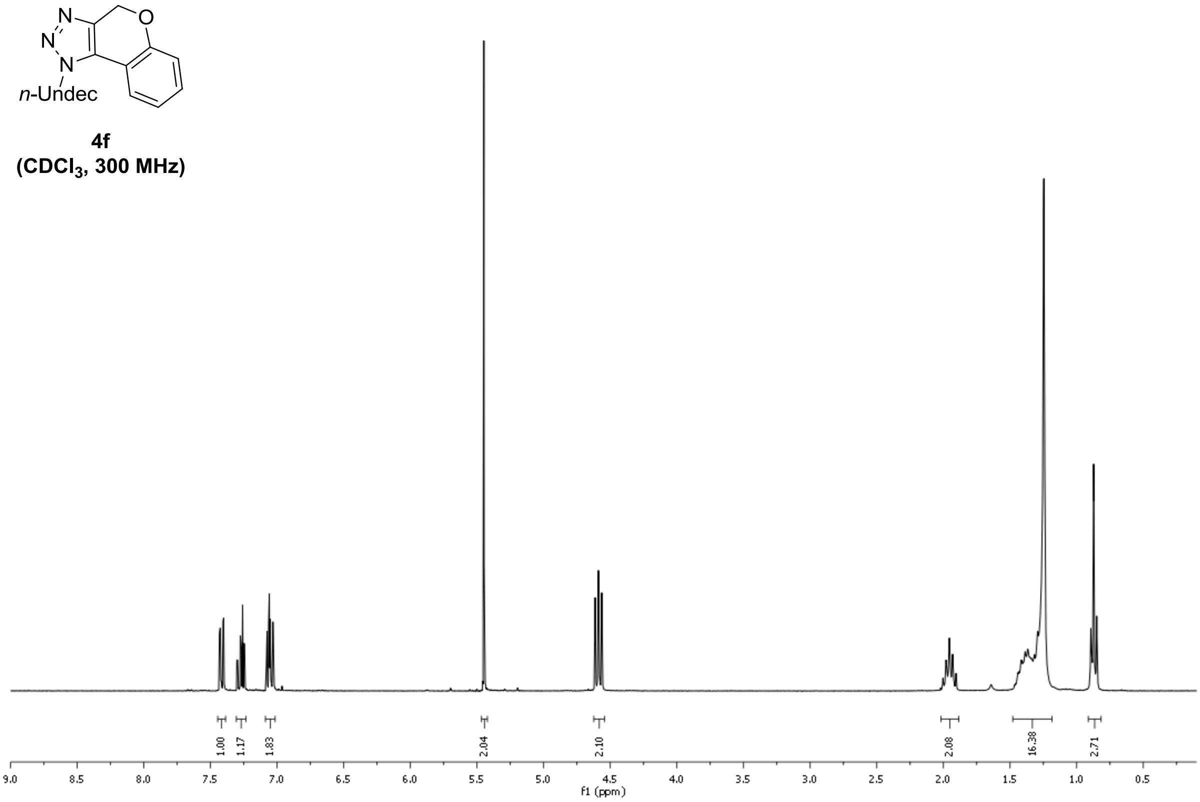

4f
($CDCl_3$, 300 MHz)

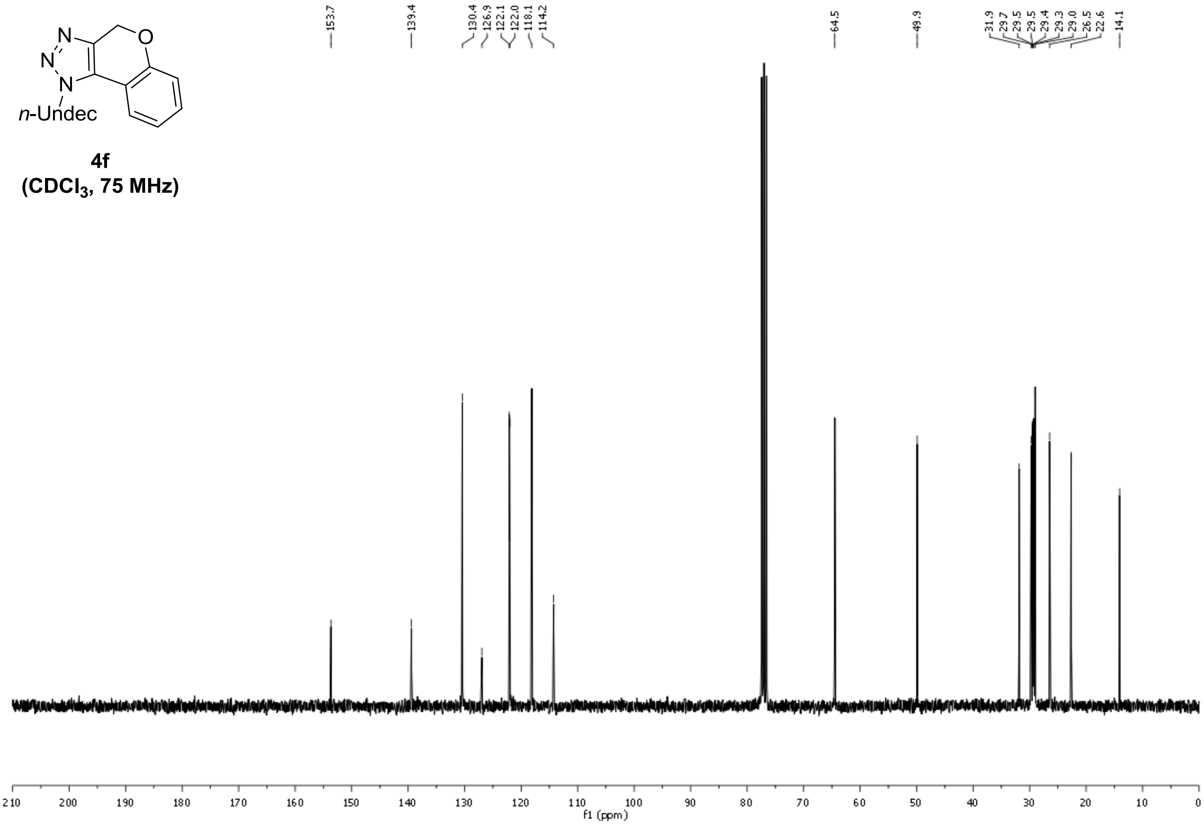

4f
($CDCl_3$, 75 MHz)

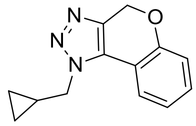

4g
($CDCl_3$, 300 MHz)

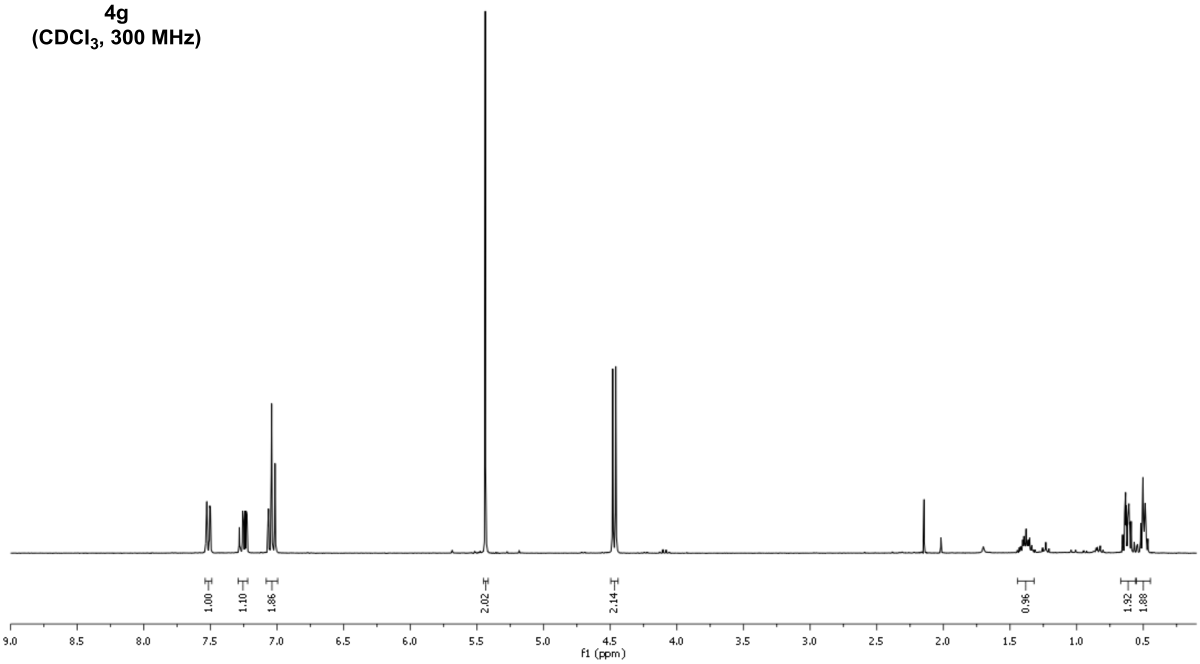

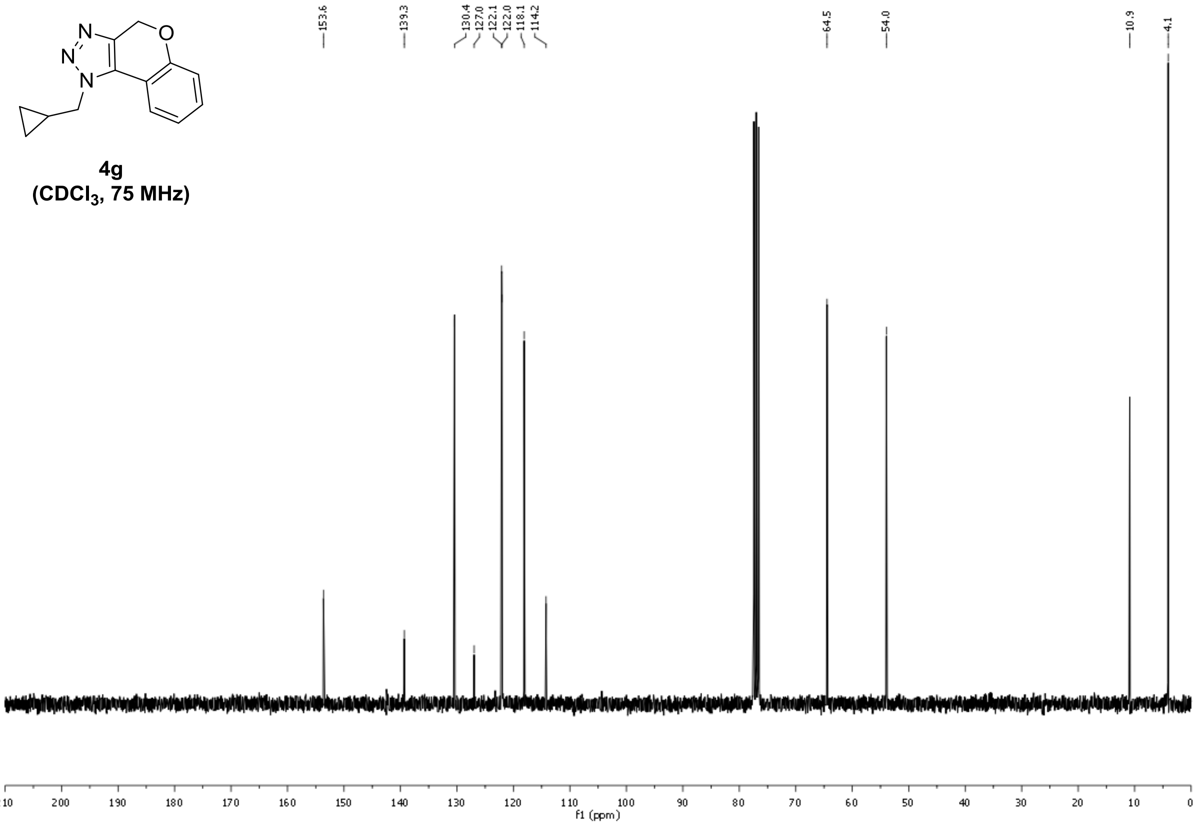

4g
($CDCl_3$, 75 MHz)

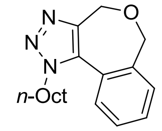

4h
(CDCl$_3$, 300 MHz)

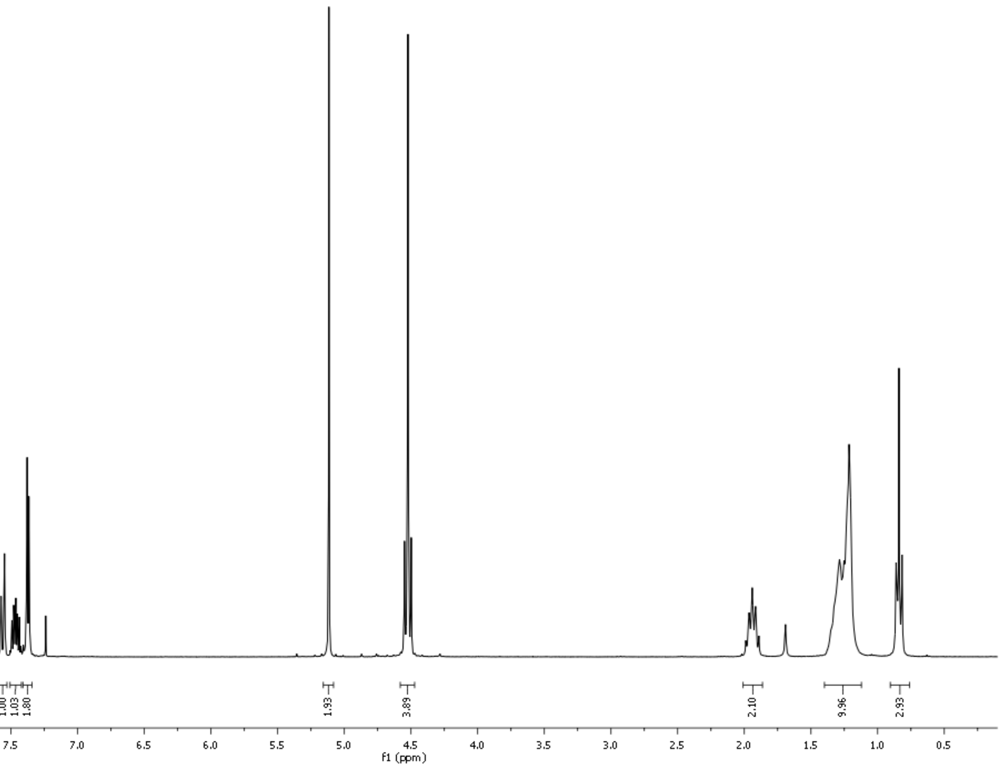

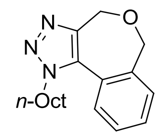

4h
(CDCl$_3$, 75 MHz)

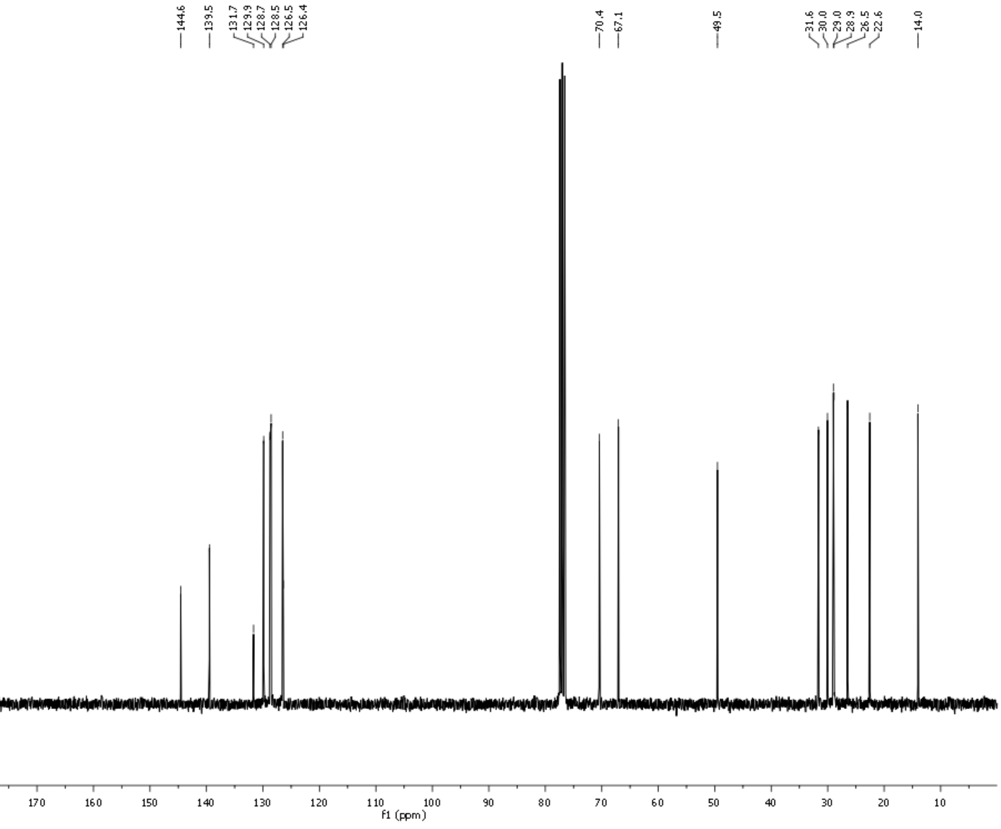

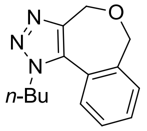

4i
($CDCl_3$, 300 MHz)

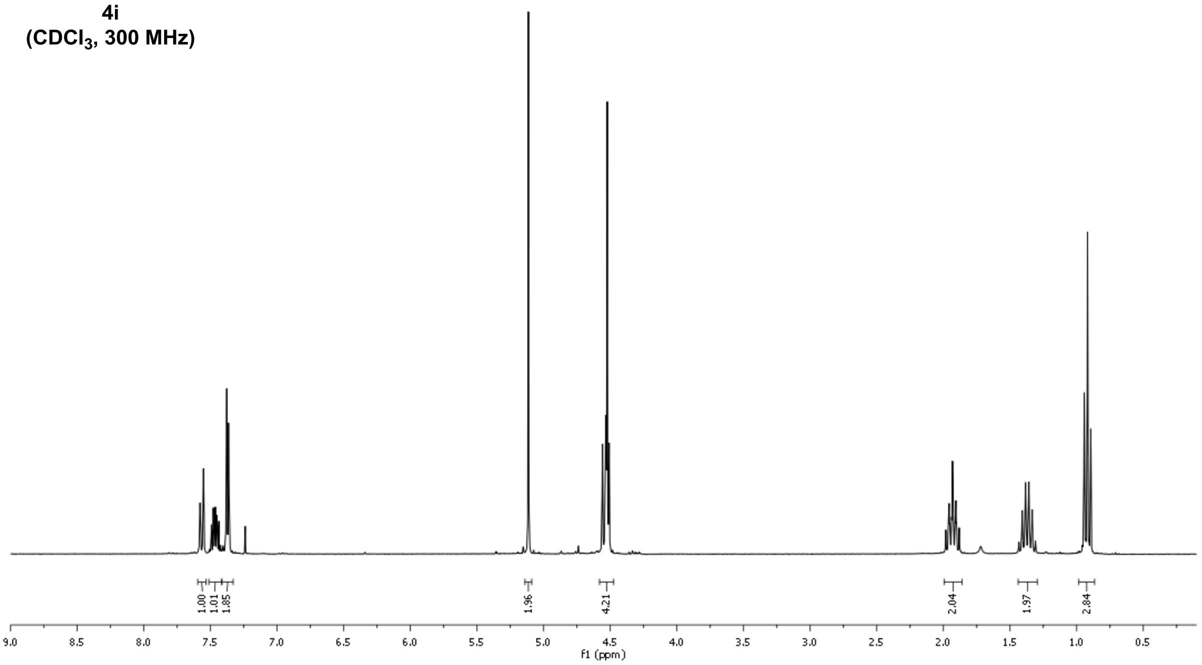

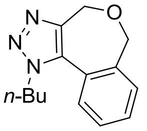

4i
($CDCl_3$, 75 MHz)

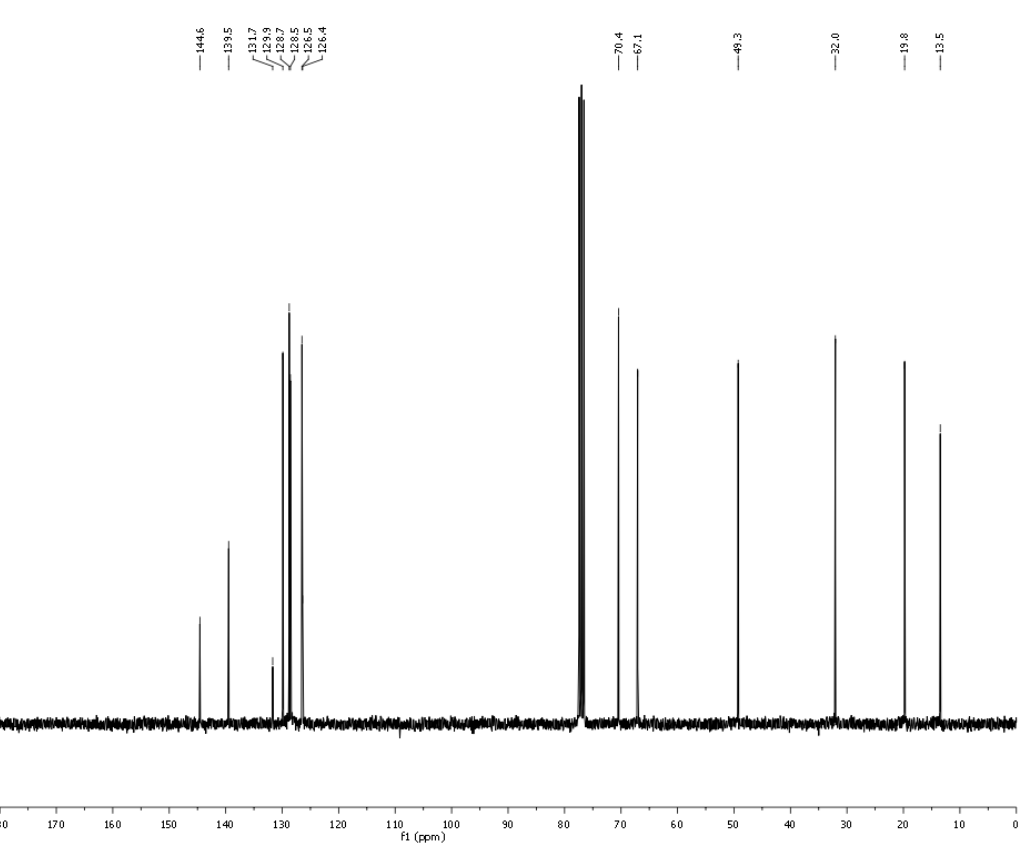

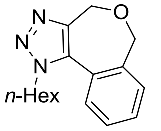

4j
(CDCl$_3$, 300 MHz)

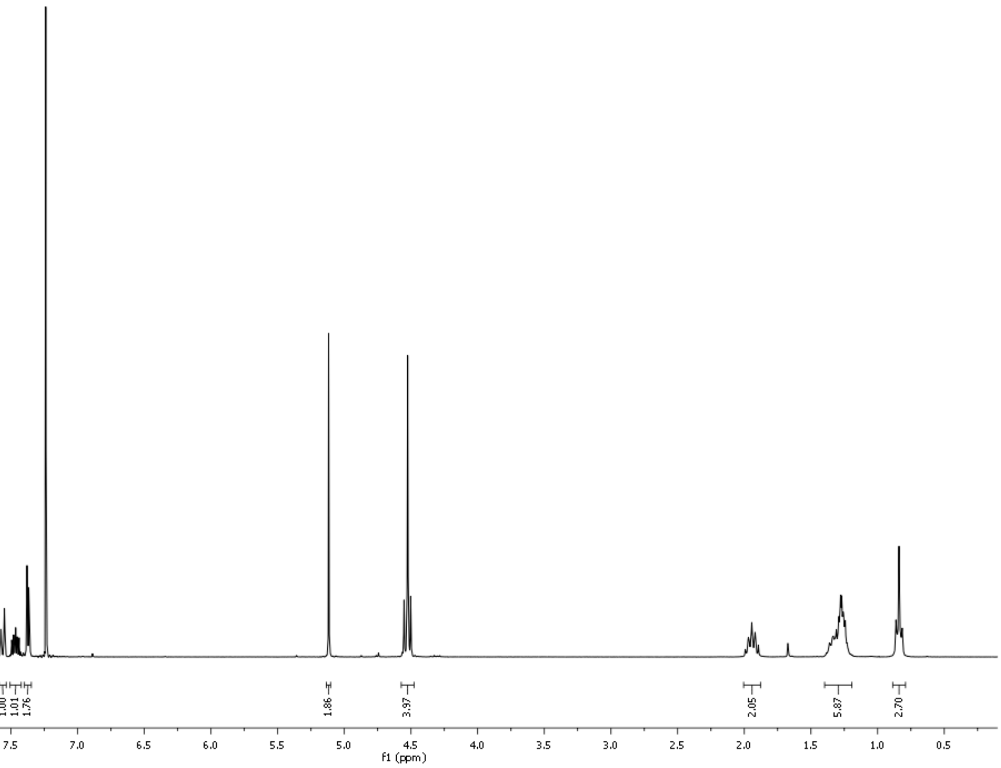

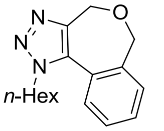

4j
(CDCl$_3$, 75 MHz)

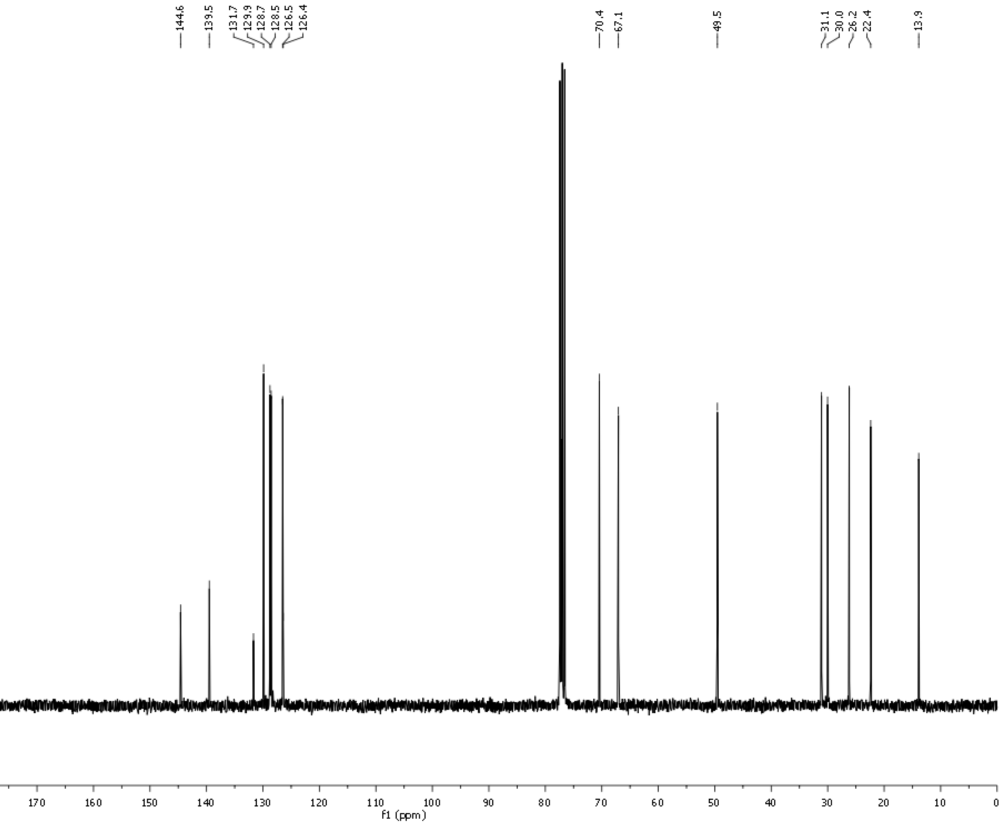

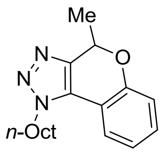

4k
($CDCl_3$, 300 MHz)

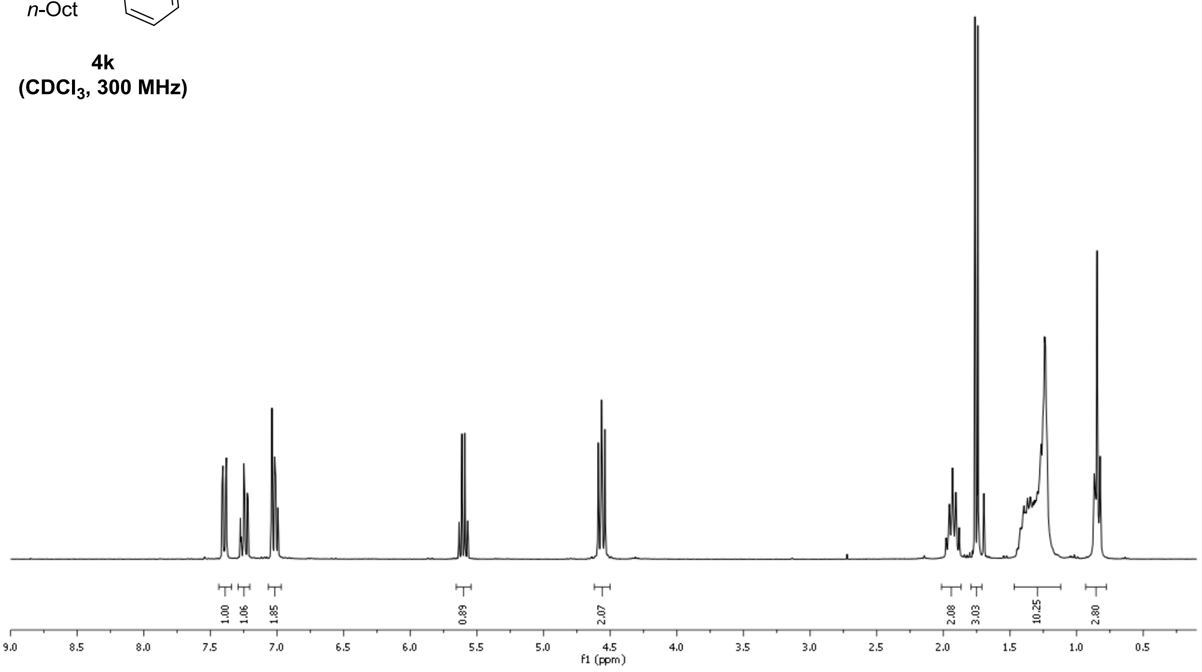

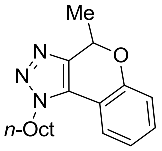

4k
($CDCl_3$, 75 MHz)

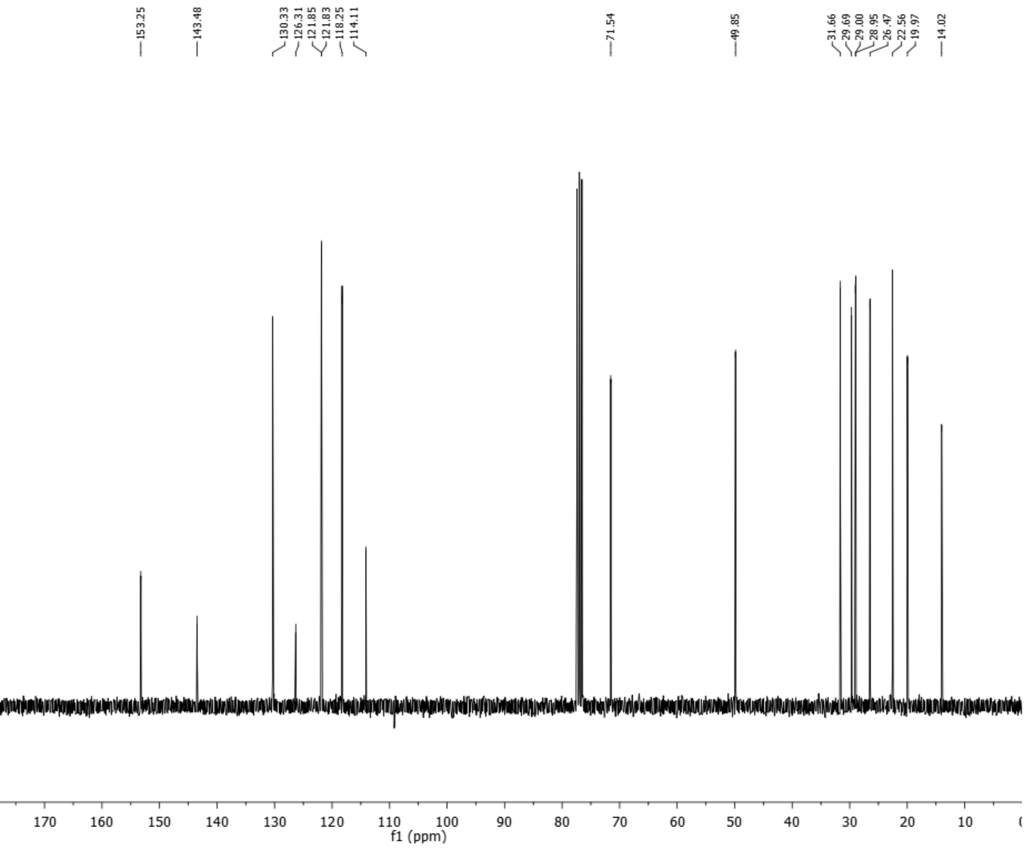

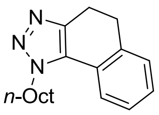

4l
($CDCl_3$, 300 MHz)

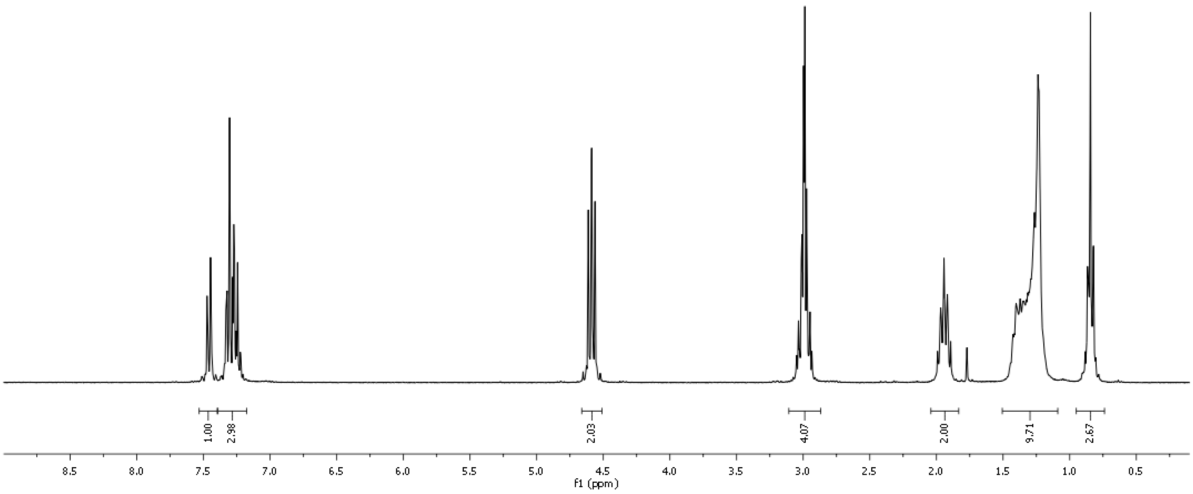

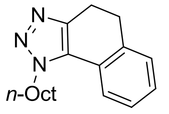

4l
($CDCl_3$, 75 MHz)

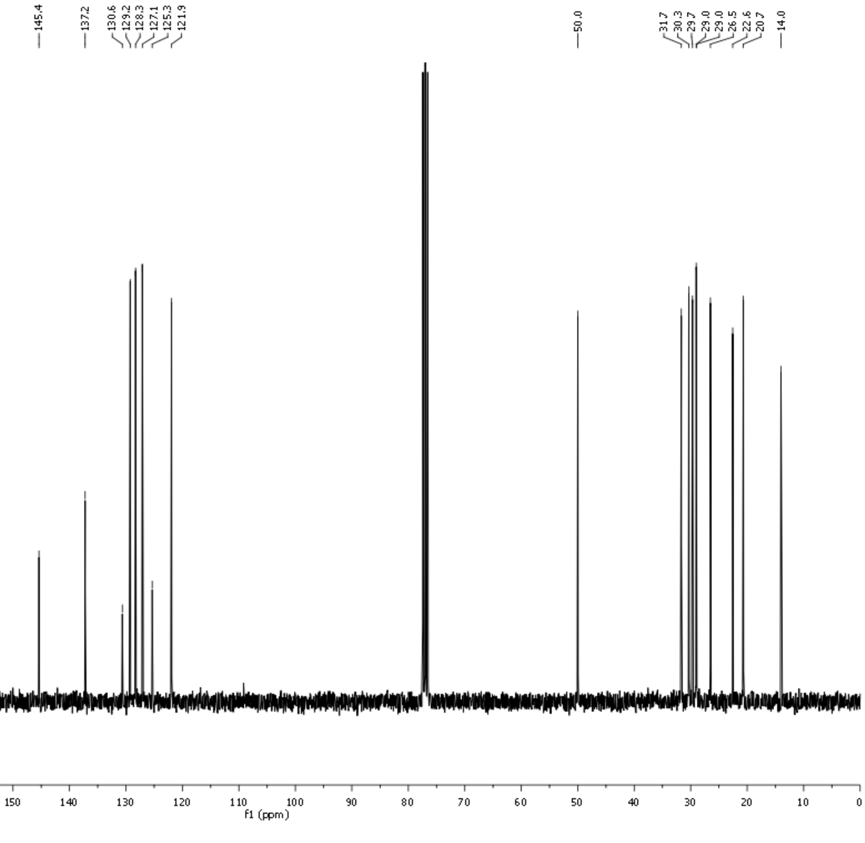

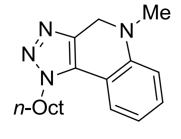

4m
($CDCl_3$, 300 MHz)

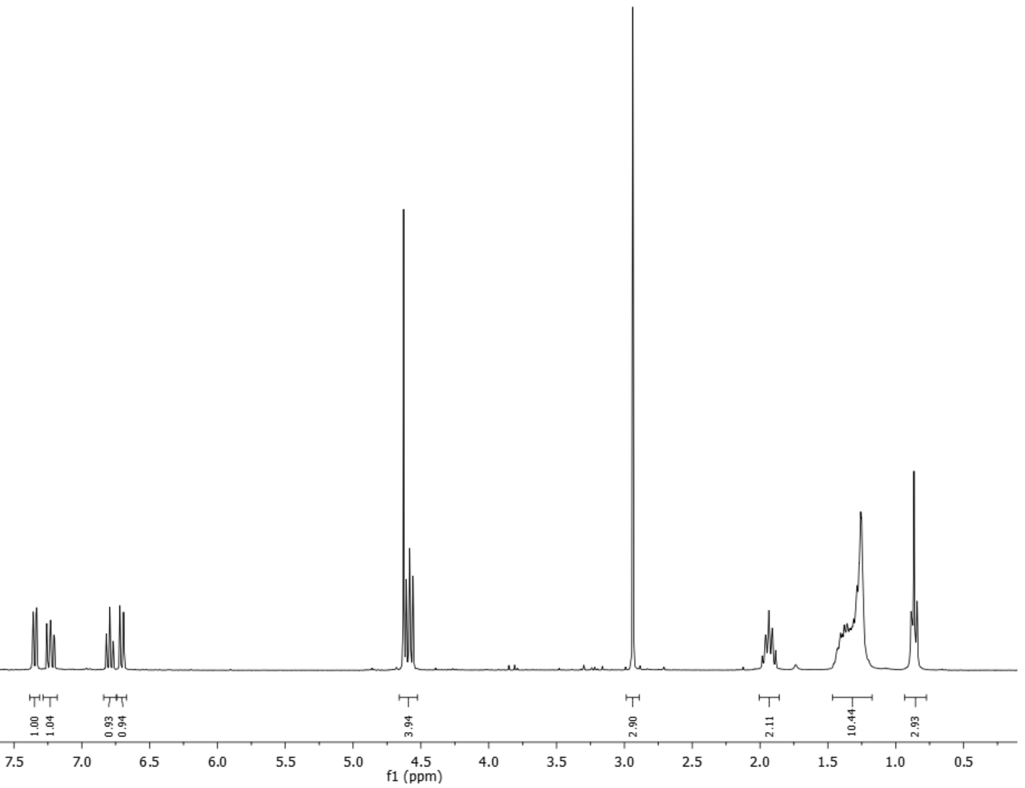

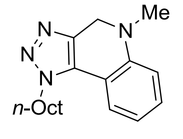

4m
($CDCl_3$, 75 MHz)

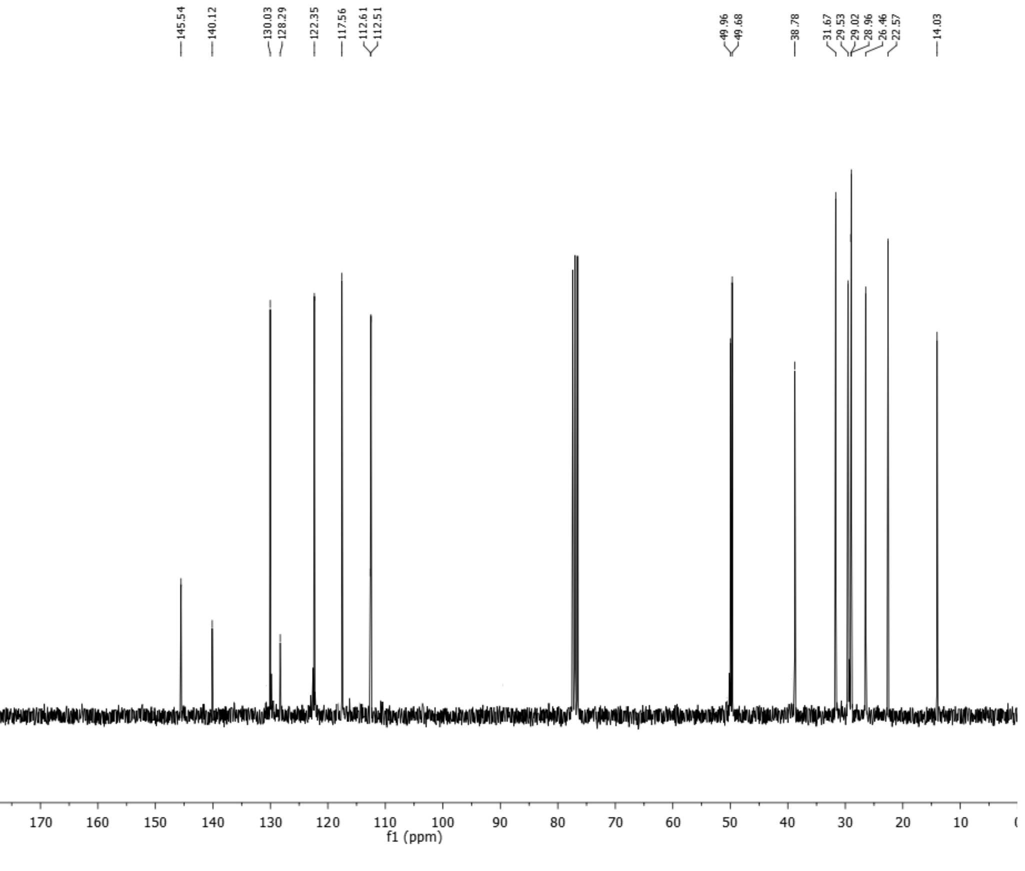

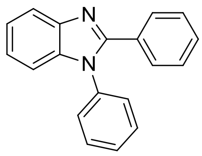

7a
($CDCl_3$, 300 MHz)

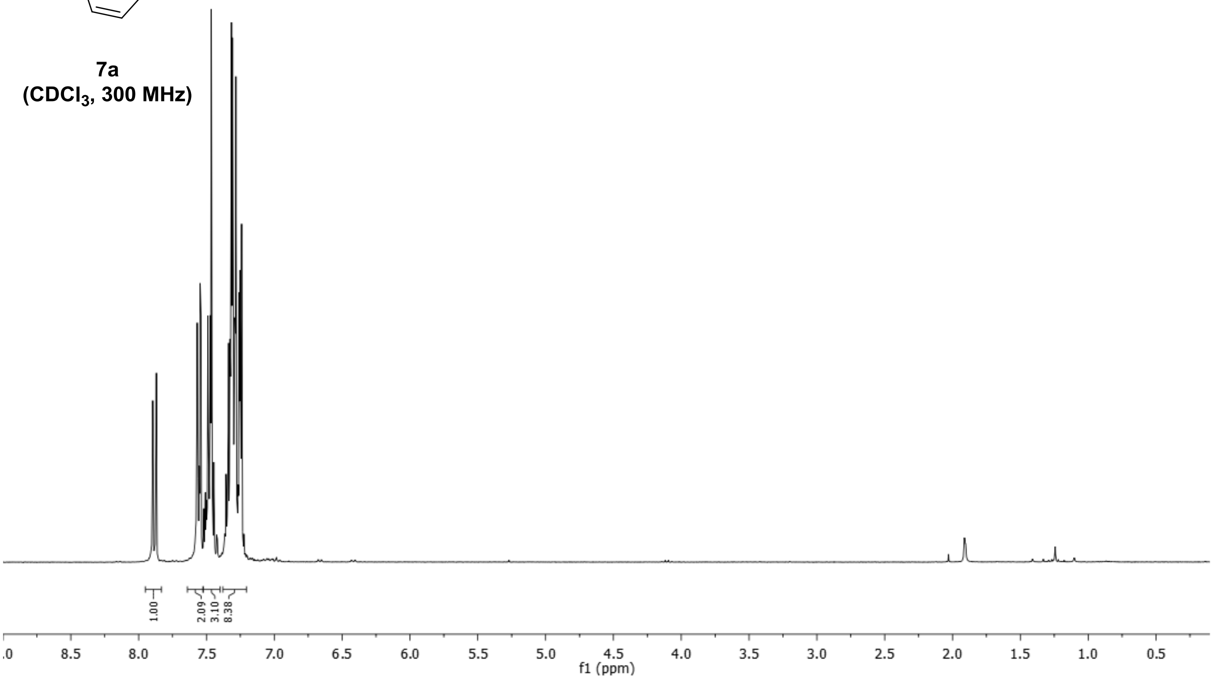

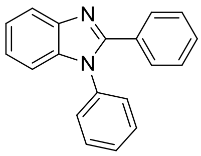

7a
($CDCl_3$, 75 MHz)

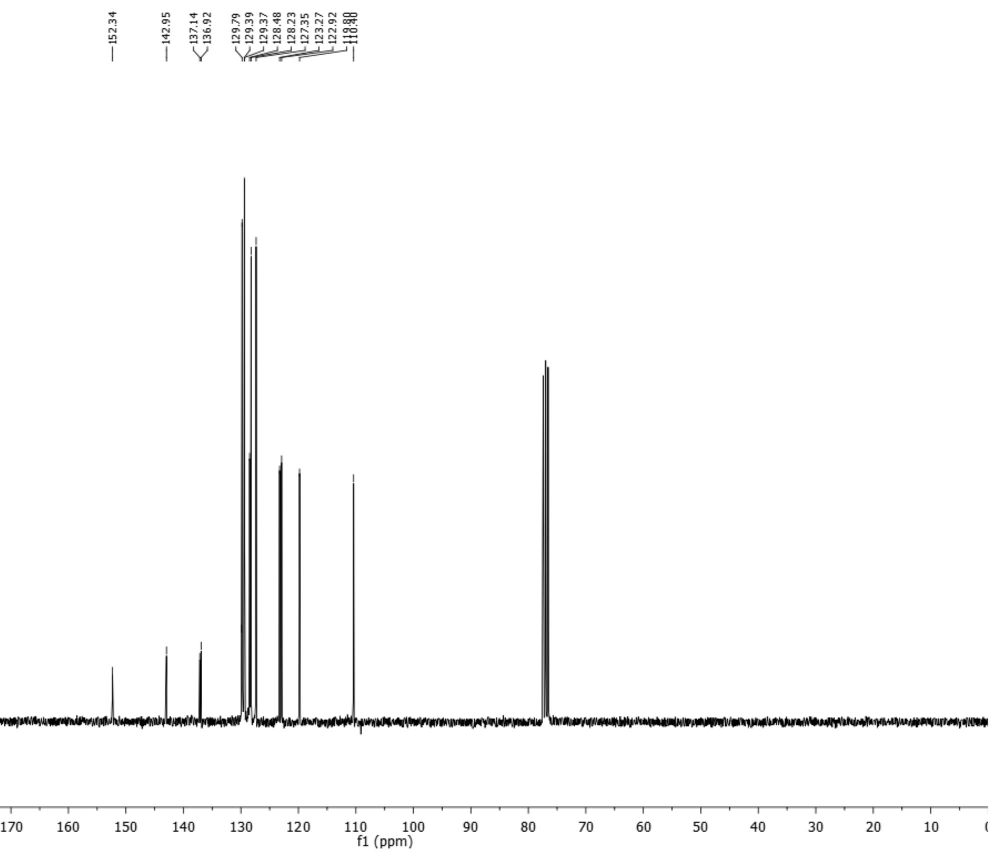

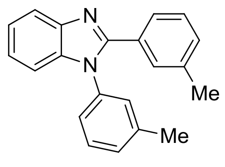

7b
($CDCl_3$, 300 MHz)

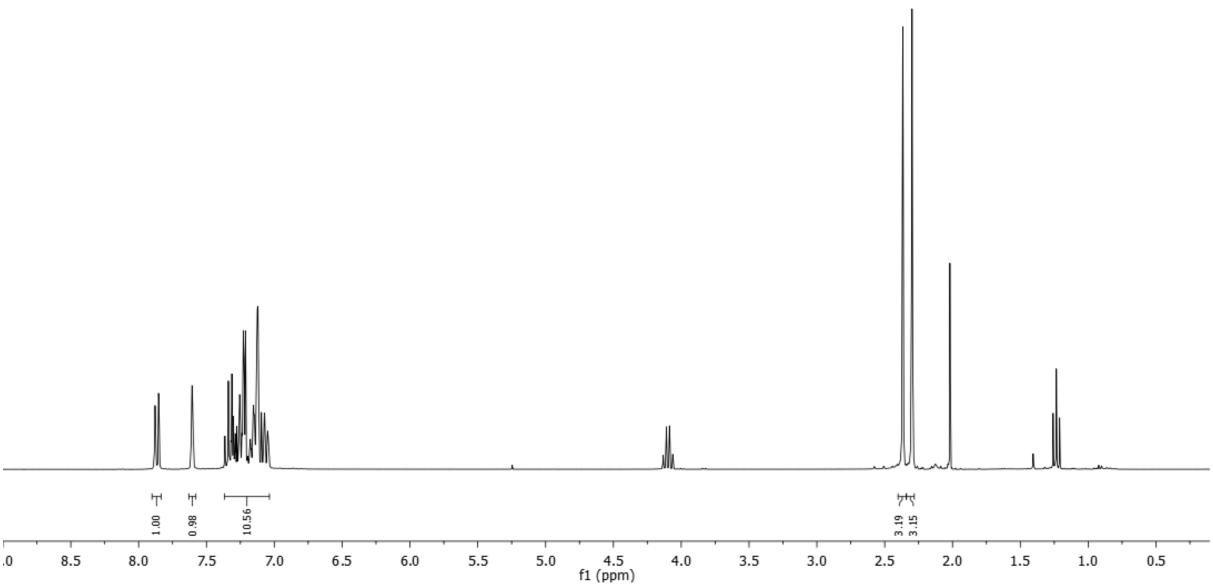

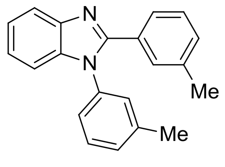

7b
($CDCl_3$, 75 MHz)

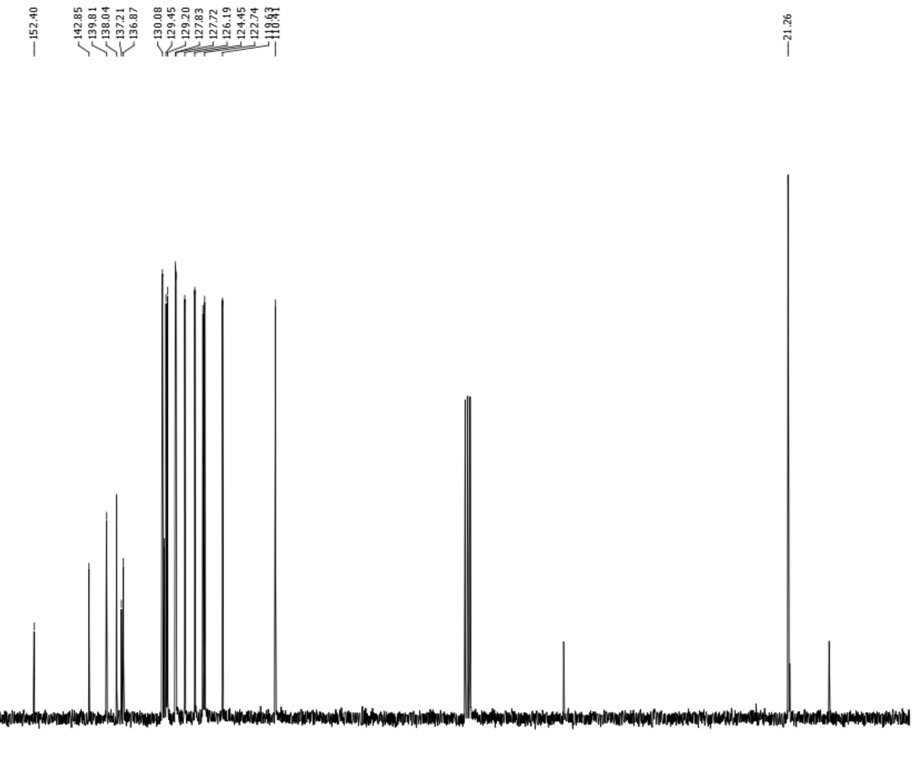

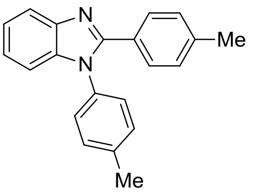

7c
($CDCl_3$, 300 MHz)

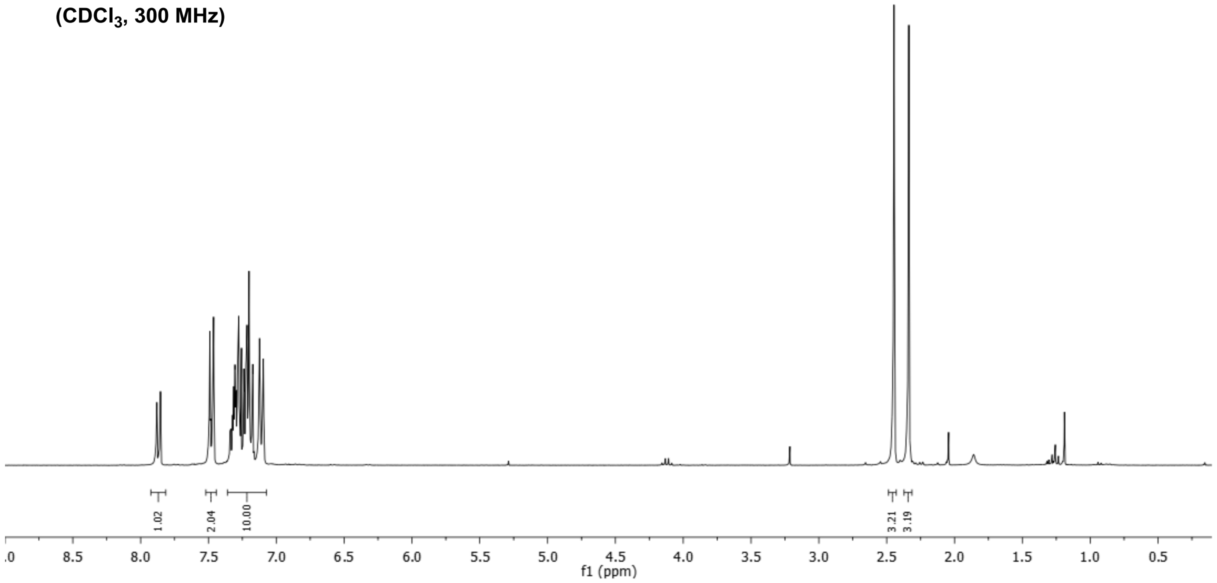

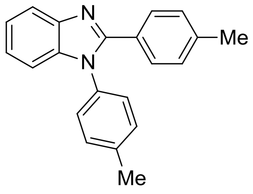

7c
($CDCl_3$, 75 MHz)

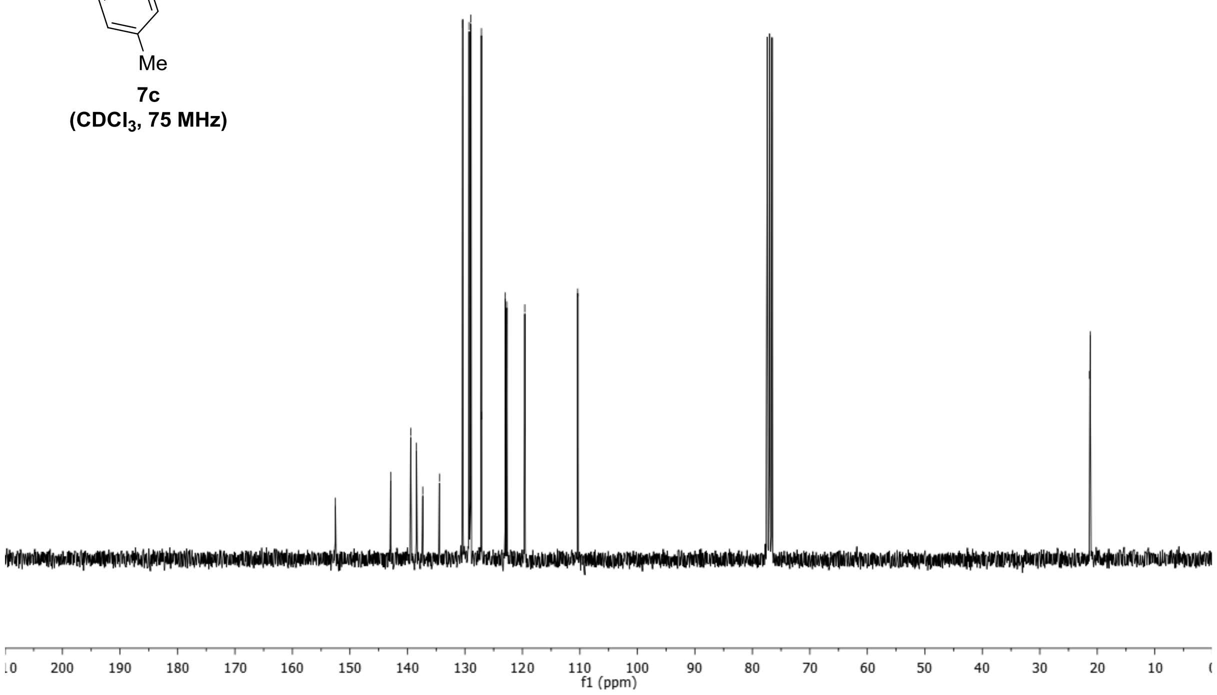

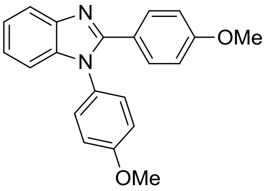

7d
($CDCl_3$, 300 MHz)

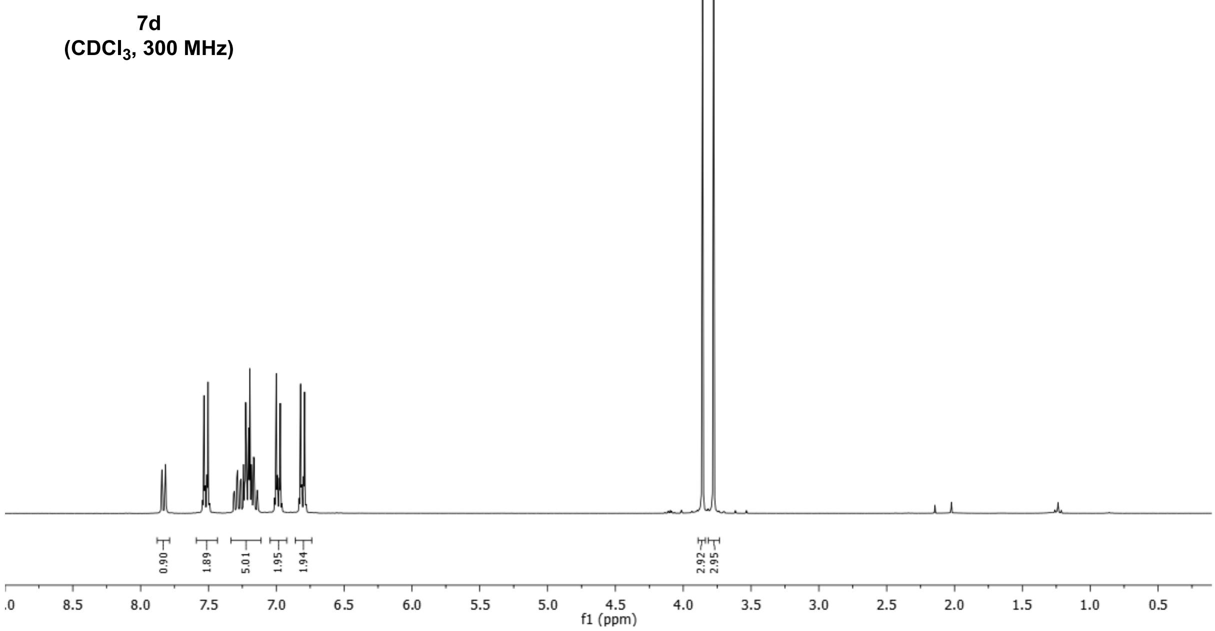

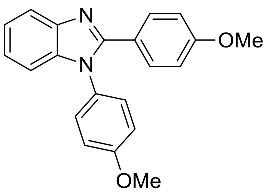

7d
($CDCl_3$, 75 MHz)

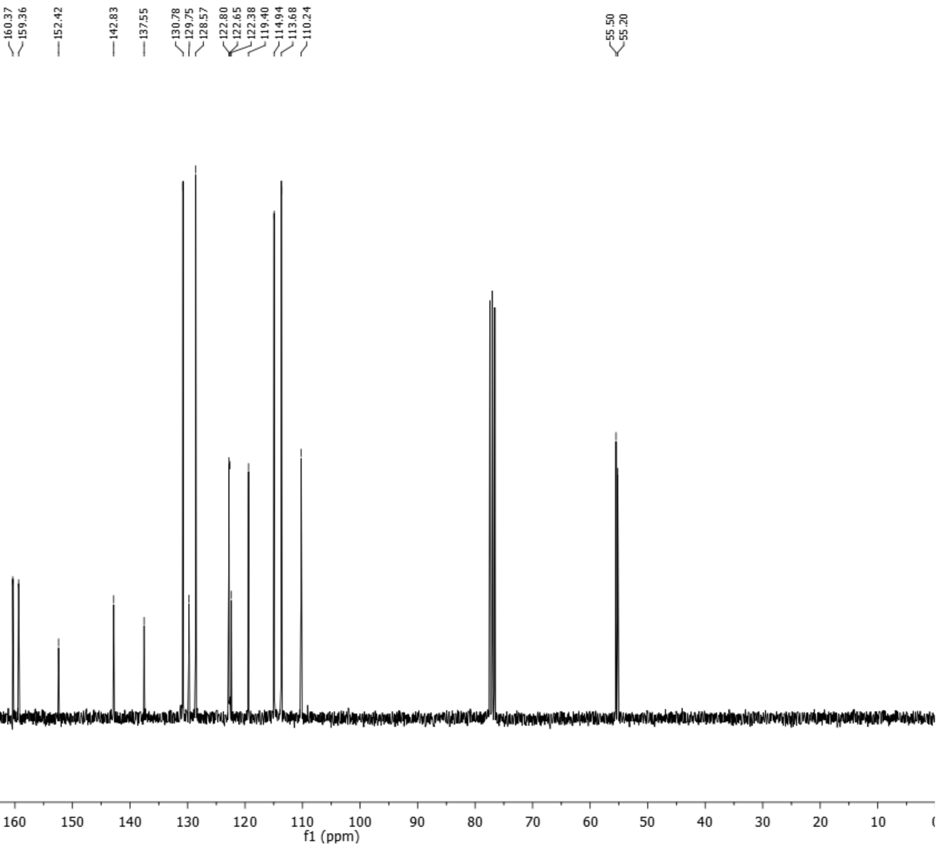

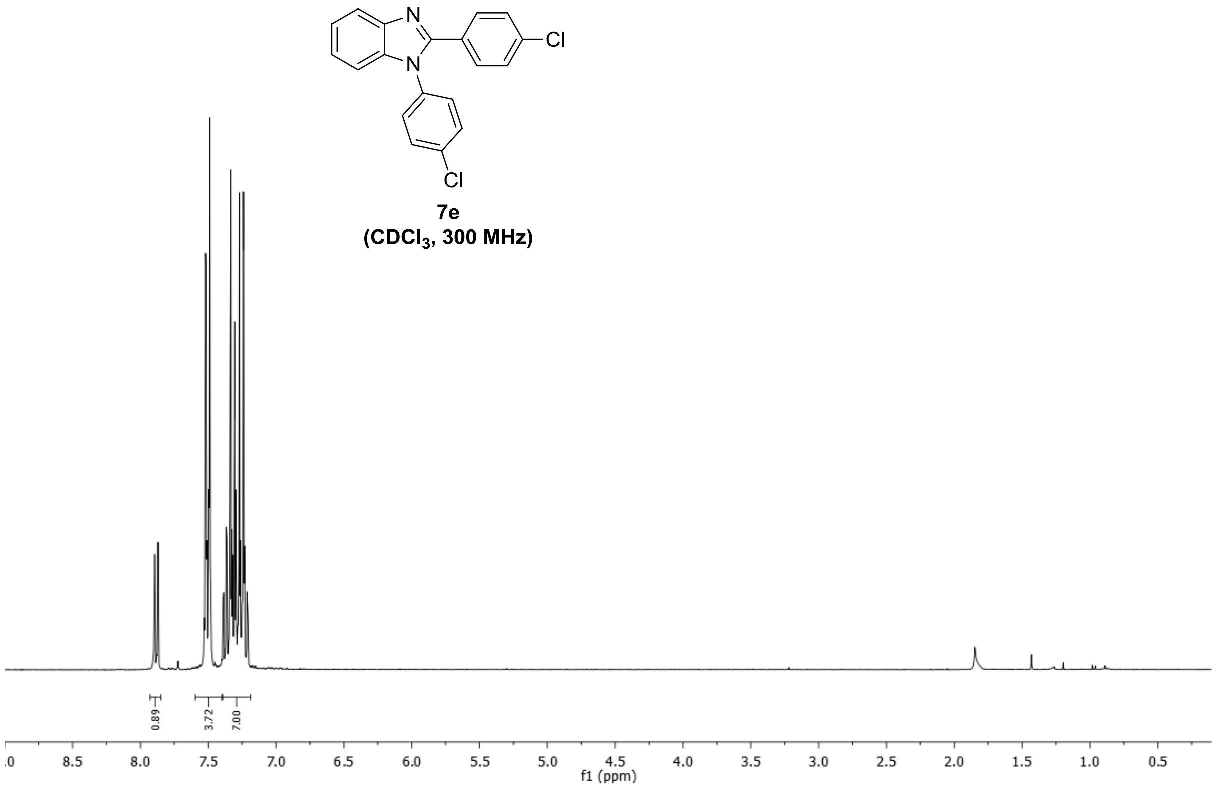

7e
($CDCl_3$, 300 MHz)

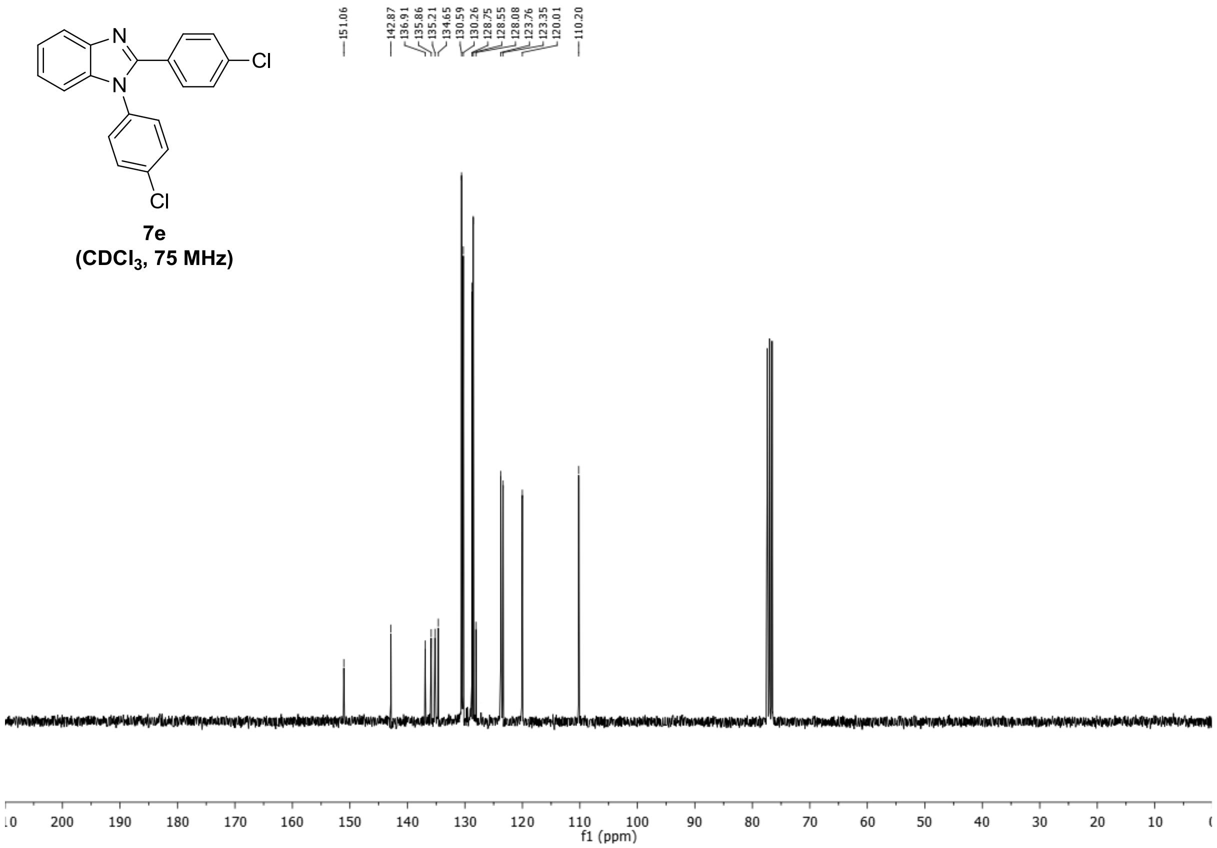

7e
($CDCl_3$, 75 MHz)

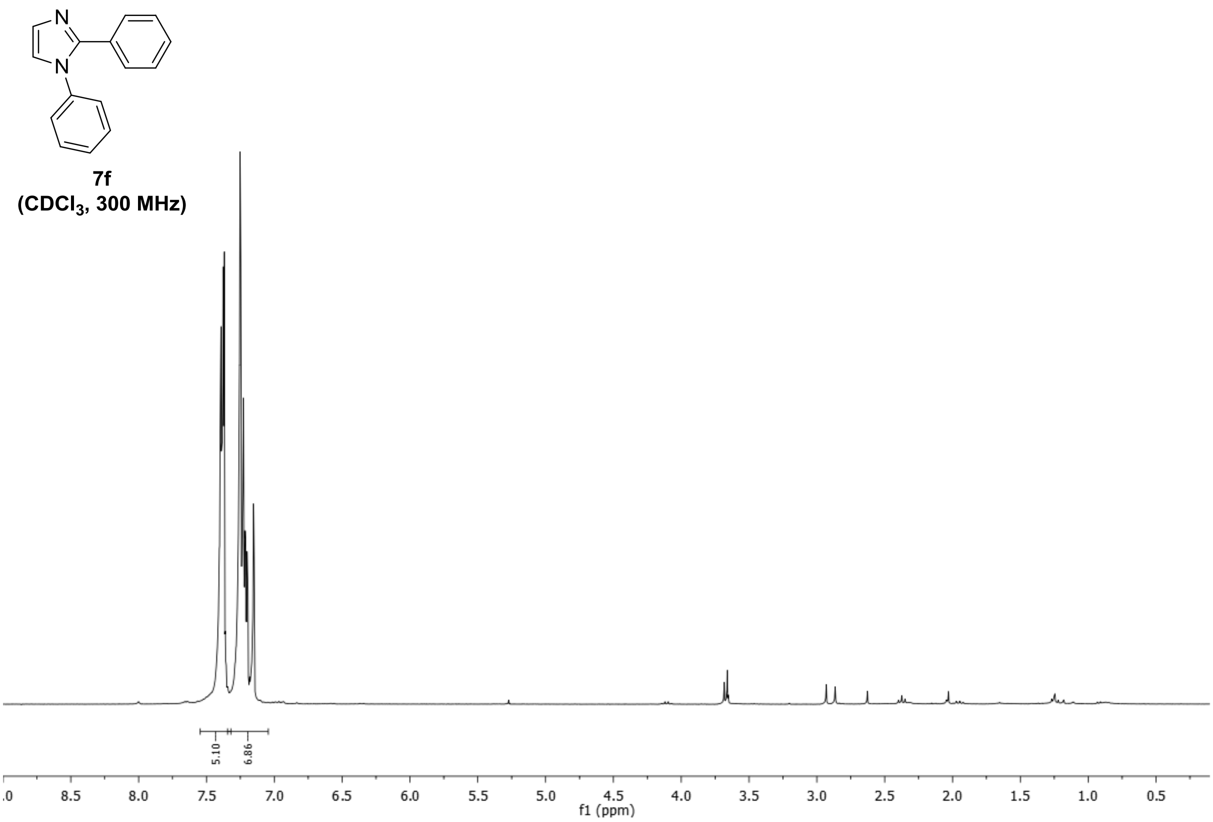
7f
(CDCl$_3$, 300 MHz)
5.10
6.86
f1 (ppm)

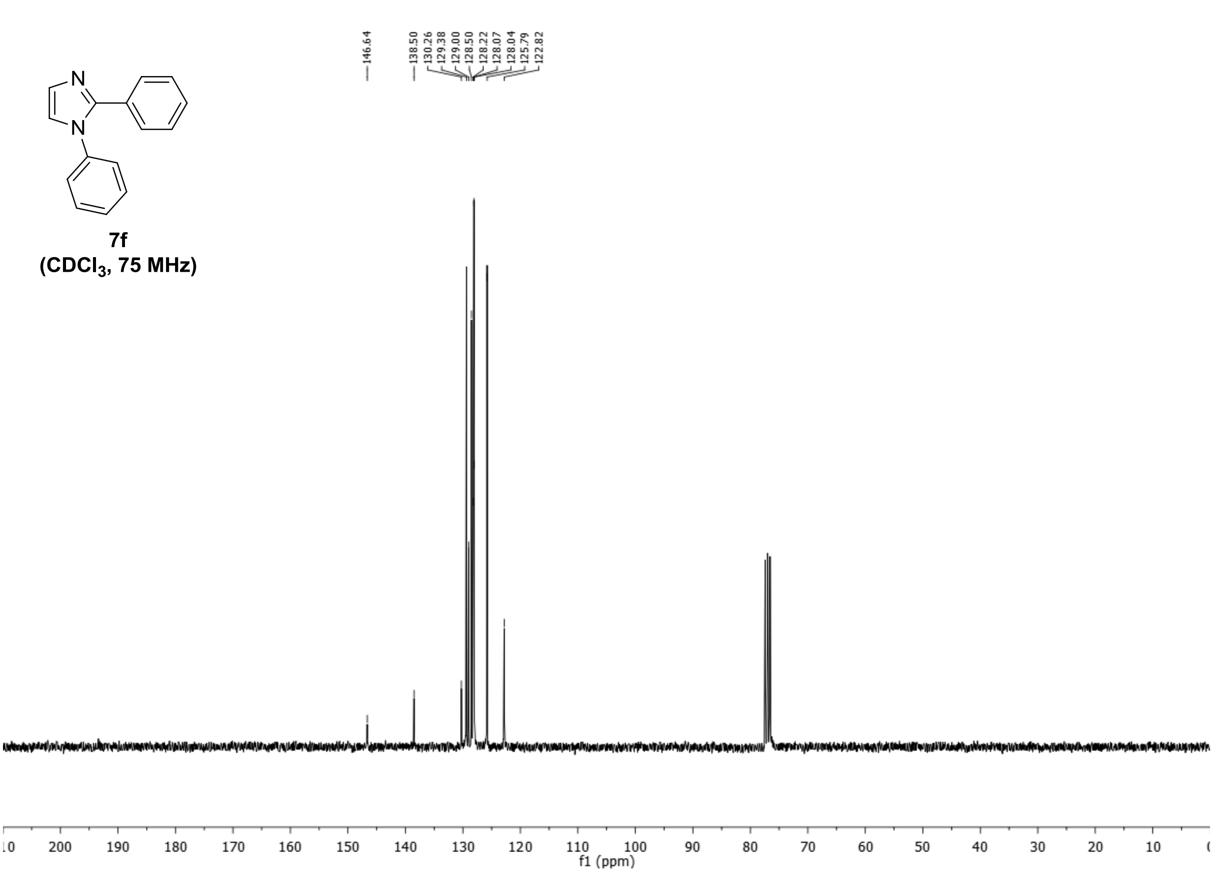
146.64
138.50
130.26
129.38
129.00
128.50
128.22
128.07
128.04
125.79
122.82
7f
(CDCl$_3$, 75 MHz)
f1 (ppm)
